# But Is It really Art? The Classification of Images as “Art”/“Not Art” and Correlation with Appraisal and Viewer Interpersonal Differences

**DOI:** 10.3389/fpsyg.2017.01729

**Published:** 2017-10-09

**Authors:** Matthew Pelowski, Gernot Gerger, Yasmine Chetouani, Patrick S. Markey, Helmut Leder

**Affiliations:** Department of Basic Psychological Research and Research Methods, Faculty of Psychology, University of Vienna, Vienna, Austria

**Keywords:** art classification, empirical aesthetics, art appraisal, liking judgments, personality, empirical methods

## Abstract

When an individual participates in empirical studies involving the visual arts, they most often are presented with a stream of images, shown on a computer, depicting reproductions of artworks by respected artists but which are often not known to the viewer. While art can of course be shown in *presentia actuale*—e.g., in the museum—this laboratory paradigm has become our go-to basis for assessing interaction, and, often in conjunction with some means of rating, for assessing evaluative, emotional, cognitive, and even neurophysiological response. However, the question is rarely asked: Do participants actually believe that every image that they are viewing is indeed “Art”? Relatedly, how does this evaluation relate to aesthetic appreciation, and do the answers to these questions vary in accordance with different strategies and interpersonal differences? In this paper, we consider the spontaneous classification of digital reproductions as art or not art. Participants viewed a range of image types—Abstract, Hyperrealistic, Poorly Executed paintings, Readymade sculptures, as well as Renaissance and Baroque paintings. They classified these as “art” or “not art” using both binary and analog scales, and also assessed for liking. Almost universally, individuals did not find all items within a class to be “art,” nor did all participants agree on the arthood status for any one item. Art classification in turn showed a significant positive correlation with liking. Whether an object was classified as art moreover correlated with specific personality variables, tastes, and decision strategies. The impact of these findings is discussed for selection/assessment of participants and for better understanding the basis of findings in past and future empirical art research.

## Introduction

“When I use a word,” Humpty Dumpty said […], “it means just what I choose it to mean- neither more nor less.” “The question is,” said Alice, “whether you can make words mean so many different things.” “The question is,” said Humpty Dumpty, “[who] is to be master, that is all.”

–Lewis Carroll, Through the Looking Glass

Viewing and experiencing art is a growing target for psychology. Because of the ability of visual art to elicit a range of psychological reactions and its importance to human action and communication, the last decade of psychological research has seen a burgeoning of studies on the appreciation and understanding of art. Among many objectives, these studies aim to investigate art taste and preferences (Chokron and De Agostini, 2000), to find cultural or interpersonal differences in art reception (Masuda et al., 2008), to assess our evaluations of beauty, liking, interest, elicitation of emotion, and, increasingly, to find corresponding areas of activation in the art-confronted brain (Jacobsen et al., 2006; Cela-Conde et al., 2013).

Using a range of images and art types, studies have shown behavioral, physiological (Holmes and Zanker, 2012; Gerger et al., 2014), and neuronal aspects of art appraisal (Pelowski et al., 2017), and have helped to differentiate the processing of aspects of the artwork such as composition, lines, colors, and more top-down meaning and context-derived response (Leyssen et al., 2012; Muth and Carbon, 2013; Jakesch and Leder, 2015; Schloss et al., 2015; Lauring et al., 2016). Results also show compelling ties to personality and expertise (Furnham and Walker, 2001; Chamorro-Premuzic and Furnham, 2004; Giannini et al., 2013), and have even uncovered compelling reactions notable for art, such as its ability to deliver awe, harmony, anger, transformation, tears, and insight (Silvia, 2009; Vessel et al., 2012; Hanich et al., 2014; Pelowski, 2015).

Most often, these empirical studies follow a design whereby a series of images—typically digital reproductions of existing artworks—are presented on a monitor, viewed for a short number of seconds, accompanied by some (liking, beauty, interest) rating(s). Often, these studies also include a pre-task instruction conveying that they will involve the viewing of “art”: for example a participant may be told that they “will be shown some works of art” or explicitly asked to “rate how much you like the artwork.” More rarely, studies might be conducted in a museum (Tschacher et al., 2012; Gartus and Leder, 2014; Brieber et al., 2015) or with framed and hung objects, where the context may imply that the stimuli are artworks (Rosenberg and Klein, 2015).

However, despite the many important advances, underlying these approaches is a major assumption that might radically impact the very basis of current findings in art-viewing research: Namely, that individuals actually do believe what they are rating is a work of art. Whether this assumption is true may fundamentally impact the underlying theoretical aspects of study interpretation; it raises questions regarding key interpersonal differences when it comes to perspectives and expectations of the participants, which are not currently explored in empirical aesthetics.

The goal of this paper is to investigate the spontaneous classification of a range of stimuli as “Art” or “Not-art” and the resulting impact of this classification on aesthetic liking ratings. We also consider the strategies used by participants in making their arthood classifications, and their personality or background characteristics, which may contribute to the propensity to classify stimuli as art, and might be accounted for when composing and assessing future research.

### A brief review: art or not art—why is our classification important?

Believing a work to be art may be key for its reception. In Western culture, art often demands a certain level of reverence, and holds implicit ties to luxury, beauty, or importance (Bailey, 2000; Hagtvedt and Patrick, 2008). Becker (1982, p. 133) notes, for example, that many may “believe art is better, more beautiful, and more expressive” than other objects. Silvers (1976, p. 443) adds, “the point of calling something art is to classify rather than to individuate it… sorting things into a class or group would be useless if everything equally warranted membership” (see also Danto, 2000). Thus, art-viewing, when contrasted to viewing other objects thought not to be art, has been empirically related to higher ratings of beauty, pleasure, and liking (Locher et al., 2001; Leder et al., 2004).

Furthermore, telling someone that something is art can lead to a higher positive aesthetic rating than when the exact same image is presented with other priming (Arai and Kawabata, 2016; Van Dongen et al., 2016). This may connect to pre-framing or pre-classification stages of current models of art processing (Pelowski et al., 2016) in which individuals prepare for viewing a stimulus, and may expect a certain level of reward—an argument supported by recent brain imaging findings that show that viewing objects expected to be art correlates to higher activation of reward and vision areas in the brain (Kirk et al., 2009b; Lacey et al., 2011; Liu et al., 2011; Kühn and Gallinat, 2012). Art has even been shown to have a contact or “infusion” effect (Hagtvedt and Patrick, 2008) on other objects—raising the assessed value of everyday items, when printed with pictures of well-known artworks.

Engaging “art” may also lead to certain modes of perception. Art viewing is often connected to adoption of a more personally detached, “aesthetic” perspective, in which perceivers attend more to stylistic and formal properties of an image, rather than its utility or content (Jacobsen et al., 2006; Cupchik et al., 2009; Kirk et al., 2009b) or employ more elaborative processing styles beyond simple object recognition (Nadal et al., 2008). Adopting an aesthetic mode, which can be primed by telling individuals that they are viewing real artworks or by being within a museum, has itself been shown to increase liking, and activation in brain areas concerned with pleasure (Di Dio et al., 2007; Cupchik et al., 2009; Kirk et al., 2009b).

Art may also involve a “transfiguration” or suffusing of ordinary objects and events with deeper meaning (Danto, 1974). This might cause viewers to look harder for significance, beyond an initial visual impression, or give more attention and veneration (Bailey, 2000). This may also allow them to enjoy even ambiguous, challenging, or negative images. For example, a laboratory study by Gerger et al. (2014, p. 175), which presented both positively- and negatively-valenced artworks as well as photographs from the International Affective Picture System (IAPS), accompanied by the information “this is art” or “this is a photograph,” showed higher liking ratings for negatively-valenced images assumed to be art. Arthood can also encourage a search for intention. Boas (1943, p. 116; see also Becker, 1982; Rollins, 2004; Kiefer, 2005) notes, for many viewers, “the fundamental distinction” of art/not-art is between controlled and random behavior. This “faith in the artist” (Parsons, 1987, p. 74) may motivate individuals to “persist in looking … when otherwise [they] would be tempted to pass onto something more meaningful.” Arthood may also be important where artworks ultimately *never* have a clear meaning, as may be the case with many Postmodern or conceptual pieces (Goldie and Schellekens, 2010). As put by Danto (1974, p. 140), rather than assuming that an object is meaningless, we may feel that it—because it is art—“is merely not about anything” (p. 142).

If objects lose art status, their evaluation may also be affected. This can be seen in studies that have considered perceived authenticity or origin in evaluation of art images. Kirk et al. (2009b) found that paintings (shown in digital reproductions), which were thought to be created by esteemed artists and borrowed from museums, as opposed to images made by a researcher, were evaluated as more appealing. Similarly, Wolz and Carbon (2014) found that paintings presented as veridical artworks as opposed to forgeries received significantly lowered estimations of quality. An fMRI study by Huang et al. (2011) found that images of Rembrandt portraits, when viewers were told that they were originals vs. forgeries, resulted in higher activation of orbitofrontal areas connected to reward (see also Noguchi and Murota, 2013). This may also be found in comparison between “real” (i.e., corporeal) art and reproductions in the laboratory. Locher et al. (1999; 2001; also Locher and Dolese, 2004) compared slide-projected or computer-based images of paintings against the originals in the New York Metropolitan Museum of Art. While evaluations relating to pictorial content or composition generally did not change between presentations, viewers, irrespective of background or training, evaluated originals more highly for interest and pleasantness, as well as found them more surprising, rare, and immediate. Brieber et al. (2014) had two groups freely view an exhibition of original photographs or reproductions in the laboratory. Original artworks were more liked, found more interesting, and viewed longer. Similarly, Brieber et al. (2015) compared viewer evaluations of contemporary paintings and sculptures in a museum exhibition against a computer-simulated version of the exhibition. Originals were found more arousing, positive, interesting, liked, and were better remembered.

These results coalesce into a pattern which suggests that the classification of an object as art is a primary factor in its appraisal (Leder et al., 2004), with key importance for interaction and neurophysiological, as well as cognitive, response. However again, unasked is the question of whether participants thought what they were viewing was art. Even the comparative lab and museum, or “original” and “copy” studies have assumed that, despite relative disliking of one condition to the other, participants were always viewing “art” in some form and responding accordingly.

### Why might individuals not classify artist-made objects as works of “art”?

Despite past approaches, it probably cannot always be expected that just because a researcher shows a picture of a sculpture or painting, it will be received as an art example. A good deal of art's history and the history of art interactions—especially since Modernism (see Becker, 1982; Dezeuze, 2005; Tröndle et al., 2014)—in fact revolve around the necessary conditions for classifying objects as art or other types of objects. Depending on the period and one's philosophical approach, individuals have used, for example, tropes of beauty, lack of function, act of making, artistic intention or control, or even sociologically driven understanding of an “Artworld” institution (Danto, 1974) as the necessary conditions for identifying art (see Hauser, 1999; Shusterman, 2000; Dutton, 2006; Wartenberg, 2006; Hagtvedt et al., 2008 for historical/philosophical discussions). These tropes have also constantly been under debate, or completely refuted, by new, challenging artworks (Danto, 1974; Becker, 1982; Richardson, 1991). In turn, with each advancement of new examples, there was a concomitant number of individuals who refused to classify these as art. This also often tied to quite negative ratings^1^. Even in the present age, among scholars, there is still debate about what is or is not art.

Stepping away from philosophy and the proclivities of the aesthetic writer or art historian, the individual empirical study viewer also probably carries an understanding of what constitutes art—to them, personally. While not necessarily logical, consistent, or in accordance with prevailing theory, it is this heuristic basis—as this individual is the one being tested—that would be expected to impact interaction in laboratory studies (Bourdieu et al., 1997; Hagtvedt et al., 2008; Lacey et al., 2011). According to a recent review by Tröndle et al. (2014), determination of arthood by the lay viewer most probably has two main axes: First, there may be a “canonical categorization” based on one's typical classification of objects. That is, an object is art because it is thought to be or to closely resemble an object that usually is classified as art. This in turn may of course take on interpersonal differences, involving the specific heuristic or philosophical approaches individuals may use when making this distinction. These may of course not be logical or immutable, but able to change at the meeting of each new object, and driven by detected cues and context. For example, a shipping container for soap may not be art because it is already occupying another class. Move it into a museum and it may become art. Although this motive can presumably be used with any art example, it may especially be responsible for many Postmodern and Modern art objects—such as Readymades or Conceptual pieces—not being classified as art, and thus often not liked by lay viewers (Goldie and Schellekens, 2010).

Second, objects may also be classified as art based on a generally hedonic reaction. In viewing an object, and when asked to make ratings, viewers may use their assessments in order to praise or “punish” a piece that conforms to their expectations or standards for a situation or object. For example, individuals may say that they do not like something that is troubling or challenging to their worldview, or even which does not resonate with their own tastes (Becker, 1982; Pelowski and Akiba, 2011). This aspect may, quite plausibly, also be used with classifying art. Individuals may feel that an object might very well typically be considered art—by experts or other viewers—however, it does not “deserve” to be so because it is in some way insufficient (Tröndle et al., 2014). As also noted in art criticism by, for example, Stecker (2000, p. 55), “it is well known that judgments that an item is or is not art are often issued to praise or disparage a given artwork, not to classify it.” While again potentially impacting all art types, this may for example tie to the well-documented dislike, by art-naive viewers—i.e., study participants—for Abstract art (e.g., Vartanian and Goel, 2004; Leder and Nadal, 2014), as well as technically bad, or challenging/esoteric examples.

Finally—especially of pertinence to laboratory studies—we note a third basis. Participants may simply not be willing to conceptually connect representations to their original art object. This “transferability thesis” (Currie, 1985; Locher et al., 1999)—which assumes that viewers are often willing to treat an image of art *as itself* the artwork—is a basic assumption of visual psychology research. However, this also has come into question due to recent results, especially those involving authenticity (see above). Finding this basis to be a major driver of classifications among viewers would obviously be a major spanner in the empirical works. Kamber (2011) further notes that the application or selection of strategies might themselves be fit into a wider context involving tastes, beliefs and past training of the individual, as well specific image qualities or art types.

### Evidence for “not-art” classifications in existing empirical art study

A review of recent empirical art viewing research does suggest emerging evidence that individuals may indeed be using one or a combination of the above strategies, raising the question of the influence of implicit art-classification. While almost never intended to be a main experimental factor, a handful of recent studies have explicitly asked if individuals believed the images that they were rating were indeed actual art—often receiving “No” as an answer.

Lacey et al. (2011, reviewed above), who contrasted paintings and similarly arranged photographic images depicting the same scenes yet without brushstrokes, found that often viewers considered the latter to not be art, although both were presented in the same context. This distinction itself may have been manly or partially responsible for the lower liking ratings and lower activation of reward brain centers. Similarly, Umilta et al. (2012) compared photographs of original slashed-canvas artworks by Lucio Fontana with simplified black and white line renderings that showed the same patterns, and asked participants to rate their sense that they were engaging with “real artworks.” While the purpose of the study was to contrast brain activation in response to the two sets of images, the authors also found lower art classification for the simplified images, which also showed lower overall ratings. Interestingly, even in the case of images of original artworks, when unknown to a viewer, these were only rated as about 65% “art,” and thus may have been a major, rather unexamined driver of the study findings. The study by Hagtvedt and Patrick (2008), in which well-known artworks (e.g., Van Gogh's *Starry Night*) or carefully-composed photographic images depicting the same scenes were printed on packaging containers, also asked participants to what degree the image was a “work of art,” finding that the photographs were significantly less art than the paintings. Whatsmore, participants did not show consensus even regarding the art status of the reproduced masterworks. The photos (which were often judged as less artful) were also again seen as less valuable and liked (see also Graham et al., 2013 for similar discussion in brain research).

Even priming participants with the information that an object is art may not always lead to definitive arthood agreement. The Gerger et al. (2014) study, also reported above, found that even with such explicit primes, when shown IAPS pictures vs. veridical artworks, participants thought the former were less artful (assessed in pre- tests), and showed less corresponding impacts on emotion and liking than in the clear veridical artwork case. These studies might also be connected to the above studies of authenticity, which also showed distinctions between both reproductions and actual paintings, as well as reproductions of “real artworks” and identical “copies”—suggesting that viewers may employ a gradient of arthood when making ratings, and may employ either classificatory or hedonic bases for their assessments.

At the same time, when we looked for studies that have explicitly investigated art classification, we could only find three examples. Most recently, Tröndle et al. (2014) considered reactions to a conceptual installation (*A Label Level*, 2009, by Nedko Solakov), composed of black marker comments written on the museum walls and commenting on the gallery's other displayed pieces. In post viewing interviews, they asked participants whether the installation was a work of art, finding positive agreement with just over half of viewers (55%). Once again, this correlated to significantly higher ratings for importance, beauty, artistic skill, composition, and even curatorial quality. As will be further assessed in the present study, they also found interpersonal differences in regards to art classification relating to age (younger individuals more frequently found the work to be art), frequency of museum visits, expectations that the exhibition would be thought-provoking, would touch all senses, and would have famous artworks, and with general appreciation of other video, performance and installation art types. However, this study was of course with only one, rather esoteric, example, and also based in a museum with physical “real” art.

Hagtvedt and Patrick (2008) also briefly describe a laboratory pilot study (p. 380) in which they showed participants a variety of images, ranging from Renaissance to Modern art, and asked them to sort them into art or not art classifications, while describing (in open-ended self-report) why they had made their decisions. Their results, although difficult to quantify systematically, suggested a mix of reasons including expressive qualities of the images (ability to transmit meaning or emotion), context, and underlying talent, creativity or skill of object maker. This study also did not report how often participants actually did classify the images as art.

Third, Kamber (2011) reported an online survey in which he presented participants with 35–38 examples which included a text-based art category (i.e., “painting”), along with a description (“Velvet Elvis: hand painted portrait of Elvis Presley on black velvet”), and, in his words, “wherever possible, a Microsoft PowerPoint image of the object.” Participants were then asked to choose whether each example was art, not art, or to check a third box if they were not sure. The findings again showed a range of positive classifications (from 6% for a pile of envelopes, to 96% for a painting). However, this study, composed in the vein of empirical philosophy, included only one example of prototypical visual art (oil painting) with the remainder being a non-systematic mix of esoteric visual examples taken from existing arguments in art criticism (e.g., Duchamp's *Fountain*) as well advertising signage, cars, nursery rhymes, poetry, photojournalism, music (John Cage's *4*′*33*″), etc. It also did not explore how or why participants were making classifications, while the survey was focused on professionals in aesthetics and the fine arts, rather than typical laboratory viewers. Thus, we might—surprisingly—conclude that the art/not art of status of stimuli used in empirical interactions remains very much a needed target for controlled, systematic research.

### Present study

The present study was designed to test individuals' classification of objects as art or not art, based on the above preliminary findings, by more systematically assessing a variety of art images, and including a range of personality characteristics, as well as motivating factors to consider the different reasons individuals may or may not see art. We were interested in assessing this topic on four levels: (1) do individuals automatically classify art images, presented in a typical laboratory study, as works of art, and if not, to what extent do they reach an “art/not art” conclusion? (2) Does the art classification impact their aesthetic appreciation of the art images, as assessed by liking? (3) What factors or strategies are used by participants when making their decisions? Finally, (4) we considered how arthood classification correlates with personality, training, or other demographic and taste factors of the viewer, in order to identify potential signifying criteria that might be important questions to ask when conducting future research.

## Methods

### Participants

The study was conducted with a final sample of 114 (80 female, *M* age = 23.2, all German speaking) psychology students from the University of Vienna, participating for course credit. The study was originally administered to 116 participants; however, two individuals did not complete all components and were thus removed from the final analysis. Participants were recruited through an online Laboratory Administration of Behavioral Sciences sign up system, were naïve to the purpose of the study, and showed low expertise and/or previous experience with making or viewing art (assessed in post-study questionnaires).

### Stimuli

**Figure 1:**
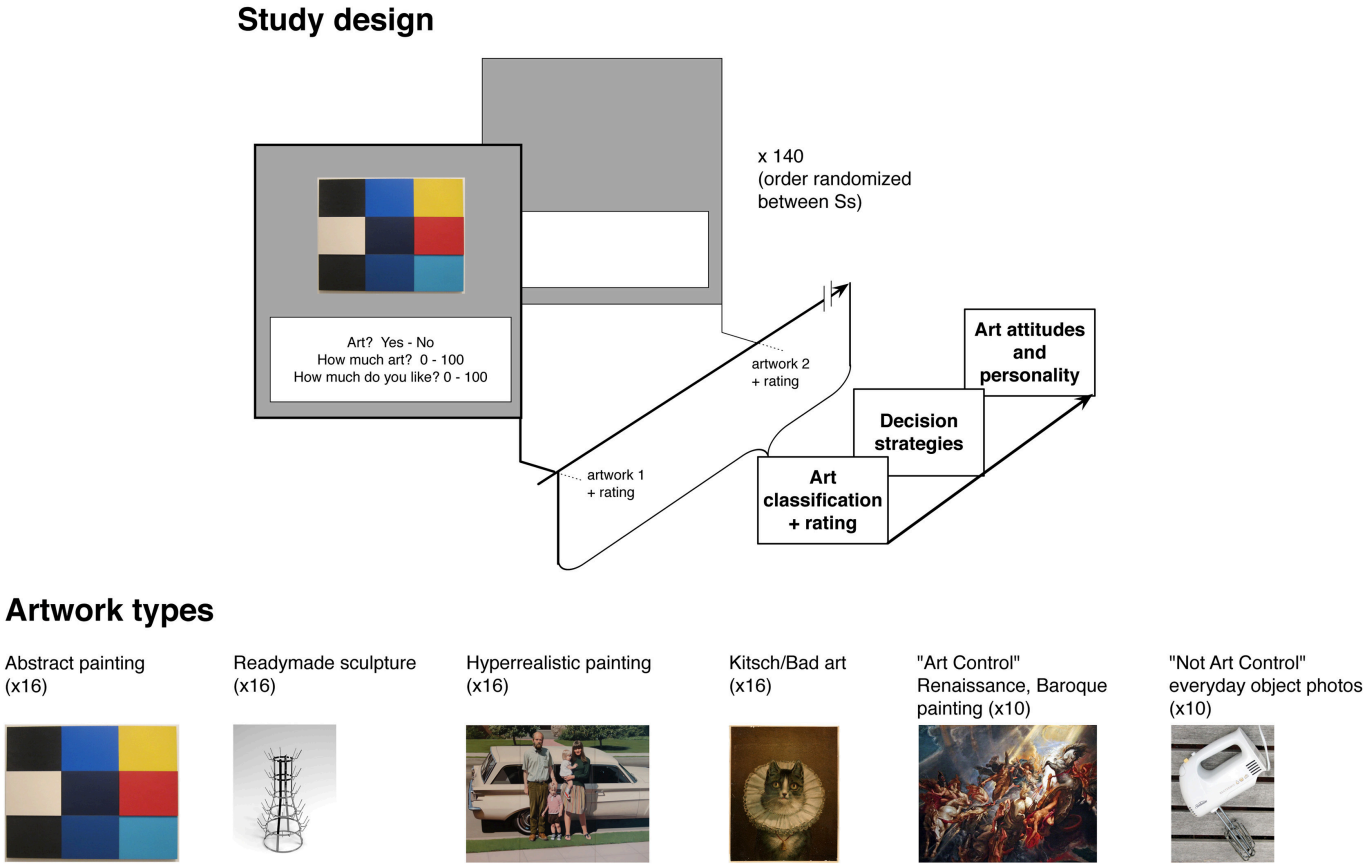
Study design and Artwork types. [Copyrights: Abstract, *Méditerannée*, oil paint on wood by Ellsworth Kelly (artist), 1952, Tate Modern, Creative Commons CC0 1.0 Universal Public Domain Dedication, Wikimedia Commons; Readymade, *Le Porte Bouteilles*, by Marcel Duchamp, 1914, photocredit filosofianetdadaismo, Flicker, Creative Commons CC 2.0; Hyperrealistic, ′*61 Pontiac*, by Robert Bechtle (artist), 1968–1969, photocredit Sharon Mollerus, Flicker, Creative Commons CC 2.0; Art Control, *The Fall of Phaeton*, Peter Paul Rubens (artist), 1577–1640, National Gallery of Art, Washington DC Patrons' Permanent Fund, image in public domain; Not Art Control, photograph of kitchen mixer, by Geocachernemesis (photographer), Flicker, Creative Commons Attribution-Share Alike 3.0].

The stimuli set consisted of 140 pictures (listed in Supplementary Material, see also Figure 1), divided into four main types (30 each), with an additional two groups of “Art” and “Not art” control images (10 each). The main set of artworks were chosen in order to systematically test the different factors whereby participants might decide that an object was or was not art. These included: (1) Readymade sculptures, which make use of everyday objects arranged or presented without an obviously high degree of artistic execution, and rely on institutional or philosophical conceptions (i.e., execution by an “artist” or placement in a museum, e.g., Danto, 1974, 1992) in order to be considered art by a viewer. They are also often categorically disliked by lay viewers (Goldie and Schellekens, 2010). (2) We also used Abstract paintings. Although fitting conceptions of 2D plastic art (i.e., executed on canvas or paper), and often hung in a museum, these are similarly often disliked by lay viewers (Leder and Nadal, 2014), potentially because they are not easily understandable or mimetic (i.e., a hedonic motive), or may not fit a classic art classification. (3) We used paintings which could generally be viewed as “Kitsch”/poorly executed. Although mimetic, they depict scenes of “low artistic seriousness” and/or suggest low technical ability. Thus, they were expected to potentially evoke a hedonic motive for classification. The “Kitsch” paintings were selected from established artists—e.g., Thomas Kinkade—while the poorly-executed artworks were taken from the archive of the museum of bad art. Last, (4) we used Hyperrealistic paintings, which depicted either ordinary objects or street scenes, seemingly without high artistic arrangement, yet with barely detectible marks of the artist (i.e., brushstrokes). These were expected to tie to awareness of making or technical quality as influencer of decisions. The “Art” Control, consisting of highly esteemed Renaissance and Baroque paintings, which were expected to be routinely viewed by participants as artworks, as well as a “Not Art” Control, consisting of photographs of everyday objects (kitchen machines, yard tools, etc.), taken with a frontal non-artistically arranged style, and which were not expected to be necessarily viewed as art. All images were collected from online archives, with uniform resolution, and with the longest dimension set at 1,000 pixels.

With the exception of the “Not Art Control” and the poorly executed paintings, all images were from respected artists. Readymade sculptures displayed evidence of a gallery context (e.g., pedestal, white walls), whereas the everyday objects were chosen to not suggest placement in a gallery or other “art setting.” Hyper realistic paintings were chosen to show some evidence of brushstrokes, given careful viewing.

### Procedure

The study was administered via online survey (Limesurvey, v. 1.92, Limesurvey.org). Upon signing in, participants were presented with a brief explanation of the project. This noted (in German) that they “would be shown a number of objects” and asked to determine whether or not they felt that these were art. Each object was then presented individually on the screen, with three questions shown below: (1) “Is the object Art?” (Yes, No, No answer); (2) “How much do you feel that it is Art?” (0–100, continuous sliding scale); and (3) “How much do you like it?” (0–100). All questions were answered via the computer mouse. After viewing and answering the questions, participants could then click a “continue” button to move to the next image. No time limit was given for answering. Order of images was randomized between participants. Each image was only shown once. Participants were asked to view the survey only on a desktop or laptop computer (not a smart phone), and to complete all questions within one viewing session. Time for completion (including post-test, below) was ~40 min.

### Post-test questionnaires

After rating all images, participants were queried directly about their classification decisions. Participants were shown a number of factors (e.g., object beauty, evidence of making, emotional evocativeness, extending from Hagtvedt and Patrick, 2008; **Table 2**) and asked to respond via 8-point Likert-type regarding how important these factors were in their decision to classify objects as art (0 = not at all, 7 = very important).

Questions were also chosen from a number of batteries with the intention of systematically measuring potential training, personality and socioeconomic/taste differences that might influence classification or appreciation of art. These included: (1) Art training and education, following Chatterjee et al. (2010) and asking participants to respond to a number of questions (i.e., “How many studio art classes have you taken at the high school level or above?”) via 7-point scale (0 to “6 or above,” see **Table 4**); (2) objective art involvement (Leder et al., 2014; e.g., “How often do you visit art museums?”, 7-point scale, “less than once per year” to “once a week or more often,” **Table 4**); (3) Art knowledge and comfort (Pelowski, 2015), e.g., “I am comfortable looking at and discussing art,” 7-point scale (1 = completely disagree, 7 = completely agree, **Table 4**); (4) General Artwork beliefs and preferences, using an unpublished list of questions from our laboratory (e.g., “the best art is difficult or challenging,” 7-point scale, 1 = completely disagree, 7 = completely agree, **Table 5**). (5) preference for different art types, including those types used in the study (e.g., “I like abstract art,” 1 = completely disagree, 7 = completely agree, **Table 6**). And (6) assessed expectations for visiting art or art museums, which were shown by Tröndle et al. (2014) to have some correlation with art classification (specifically with “to have my thoughts provoked,” “to be part of the exhibition with all my senses,” and not “to see famous artworks,” 1 = completely disagree, 7 = completely agree, **Table 7**).

In addition, we assessed general aspects of personality via (7) the Big Five Questionnaire (German 10-item short version (Rammstedt and John, 2007), (8) Need for Cognitive Closure (16-item German version, Schlink and Walther, 2007) identifying desire for firm belief on a given issue, as opposed to ambiguity (Leone and Chirumbolo, 2008); and (9) the Creative Personality Scale (Kaufman and Baer, 2004; all **Table 8**). (10) Finally, we administered the complete cultural taste and attendance questionnaire used by Hanquinet (2013) in her study of types of art viewers or attendees to museums. This asks participants to respond via Likert-type scales to a number of activities or cultural media involving tastes in art, music, and books as well as attendance at both low and highbrow activities (e.g., viewing TV, attending the ballet, respectively), and involvement with creative activities (e.g., participation in playing music; **Table 9**). Participants were also queried regarding basic demographic information (age, sex, occupation, and nationality). Question groups and order of individual questions within the batteries were randomized between participants.

### Ethics statement

This study was carried out in accordance with the recommendations of the Ethics Committee of the University of Vienna. All subjects gave informed consent in accordance with the Declaration of Helsinki. The protocol was approved by the Ethics Committee of the University of Vienna.

## Results and discussion

Results are reported in the following order: (1) We discuss the basic results of the artwork classifications and (2) the relation between art classification and liking. We then consider (3) the various strategies used by participants when making classifications and how these relate to higher or lower likelihood of finding objects to be art. Finally, (4) we consider the personality factors in regards to how these correlate with classifying the images as art. An alpha of 0.05 was used for all assessments. Because this study involved a large number of, often exploratory, measures, we also integrate results with a discussion of previous literature where relevant. This is followed by a brief summary in the conclusion.

Due to the instance of observed skew in the visual inspection of some study items and violations of normality of distributions (assessed via Shapiro-Wilk tests), non-parametric analyses were used for all reported correlations, which are sensitive to normality issues. (However, see Field, 2009 or Altman and Bland, 1995 for arguments against the veracity of normality testing and suggestion that even data that violates normality assumptions can be reliably used for parametric tests, especially with largish samples). Inspection did show that all data appeared to follow a linear relationship in the correlations. The non-parametric assessments employed Kendall tau-b, which is argued to provide a more accurate and conservative test than similar Spearman rank-order (Howell, 1997) and is also largely more conservative than parametric Pearson product-moment. Note however, that Pearson product-moment analyses gave highly similar results. Because many readers may utilize parametric assessments, and for the sake of comparison, these are reported in Tables in the Supplementary Materials. For repeat measures comparison of group scores, we employed ANOVAs, with Mauchly's test of sphericity reported in the text. In case of violation, Greenhouse-Geisser corrections were applied indicated by the corrected degrees of freedom. For exploratory data reduction we employed Principle Component Analyses (described below).

Note also, due to the exploratory nature of this study and large number of factors, all significance levels for correlations coefficients are reported uncorrected. This was based on the argument for best practice in exploratory research (Rothman, 1990). We urge the reader to be mindful of this when making inferences.

### Do viewers automatically classify artworks as “art”?

**Figure 2:**
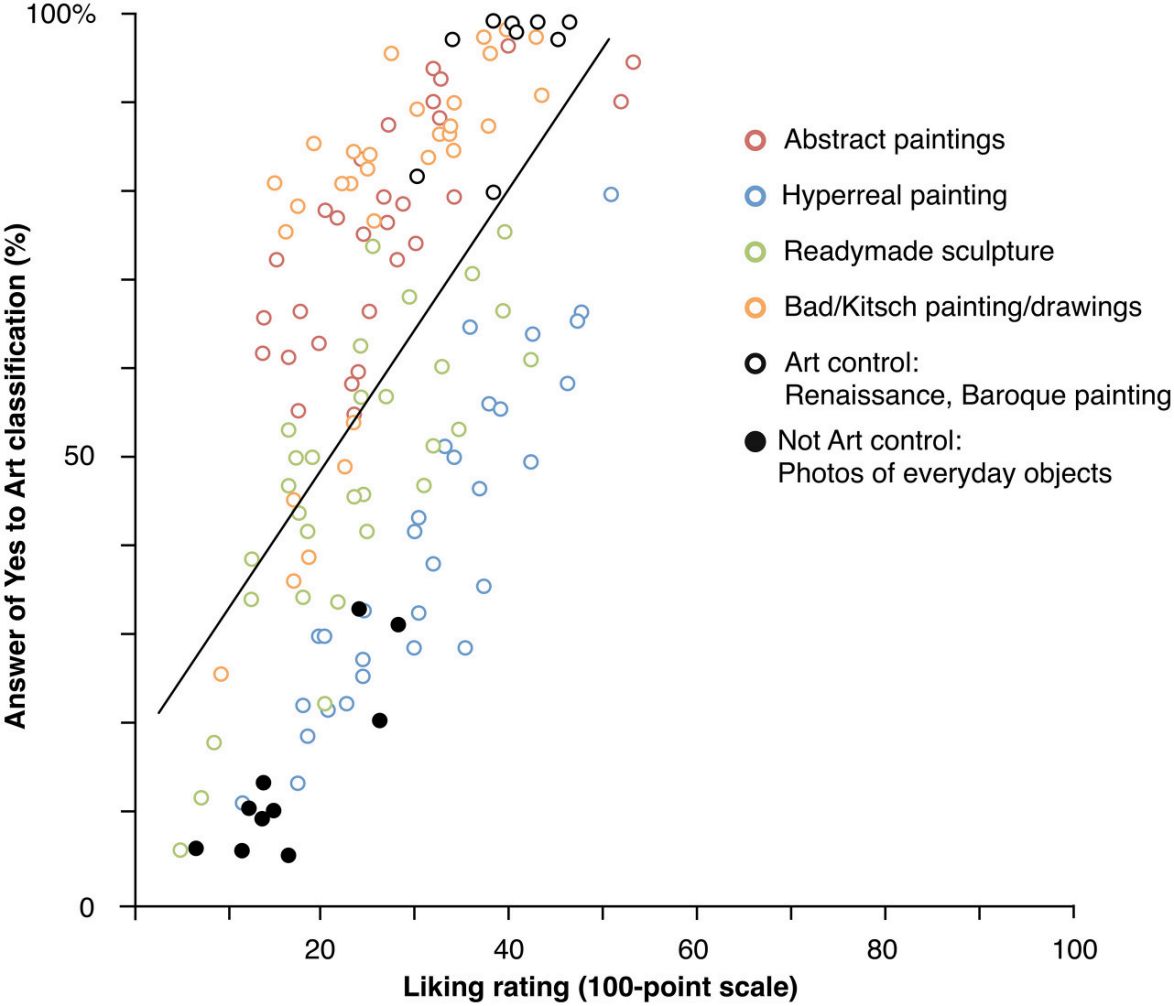
Left: Average percentage “Art” classifications and liking ratings for each individual image, divided between art types. Right: comparison of 100-point “How much is it art” ratings between objects classified as art or not art, within each image type.

Results of the classification of images as art or not art, as well as evaluation for liking, are shown in Table 1. See also Figure 2, which displays each artwork as a dot, with the y-axis representing the percent of participants who found it to be art, and the x-axis representing average liking ratings. To assess the classifications, the 100-point “How much is it art” scores were averaged for all images within each of the six object types (Abstract, Readymade, Kitsch, Hyper realistic; as well as Renaissance/Baroque paintings and everyday objects), for each participant. The individual scores for each art type were then used in the following analysis. A similar analysis was also conducted for the 100-point liking ratings. For the binary Art/Not art answers to each image, the percentage of positive “Art” answers were calculated within each art-type by dividing the number of positive answers by the total number of artworks within the set.

**Table 1:** Classification of images as “Art”/“Not-Art” and Liking ratings (across participants).

	**Classification as “Art” (% Yes)**		**How much is it art? (0–100)**	**How much do you like? (0–100)**	**% participants who answered 100% “Art”**
	**M (SD)**	**Range**	**M (SD)**	**M (SD)**	
Abstract paintings	76.0 (12.6)	3.4–100	45.7 (10.0)	26.7 (9.5)	16.8
Readymade sculptures	47.8 (17.4)	0–100	29.5 (10.2)	23.1 (9.7)	4.4
Hyperrealistic paintings	40.8 (17.8)	0–100	27.1 (10.8)	31.0 (10.3)	6.2
Bad/Kitsch paintings	77.7 (19.7)	10–100	52.7 (17.0)	27.1 (9.1)	19.5
Control “Art” (Renaissance/Baroque paintings)	95.0 (7.3)	70–100	77.8 (6.1)	39.0 (5.2)	72.6
Control “Non art” (object photos)	14.9 (10.3)	0–100	12.0 (5.7)	16.5 (7.0)	6.2

*Results based on N = 114 (80 female, M age = 23.2, native German speaking) psychology students from the University of Vienna*.

First, as can be seen from these results, across all of the art types, participants often did not feel that all examples were works of art. Further, they showed rather large differences between categories. Looking at the percentage scores for classifying the objects as art, the bad/kitsch paintings and drawings showed the highest rates of art classification (77.7%, averaged across all participants), followed by Abstract painting (76.0%), and then by the Readymades and Hyper realistic paintings, both of which showed classification rates below 50%. On the other hand, the Renaissance/Baroque control paintings showed higher rates (95%), while everyday objects were classified as art in only 14.9% of cases.

The 100-point “how much is this art” answers largely followed the same patterns as the above percentages: The highest scoring was found for the Renaissance paintings (*M* = 77.8), the lowest ratings were found for the photos of everyday objects (12.0), and the other art categories ranged from ~27 to 50. Significant correlations were found between the 100-point scores and the above percentages for all art types (^τ^B range = 0.37–0.56, all *p*s < 0.01). Assessment of correlations, regarding the percentages of art classifications for the main types of objects were also significant between all types (^τ^B range = 0.23–0.58, all *p*s < 0.01)—i.e., classifying one type of image as art correlated with finding another type to be art. Similar results were also found for the 100-point scores (^τ^B range = 0.29–0.60, all *p*s < 0.001).

Note also Figure 2, right side, which splits the 100-point ratings for art types between objects selected via the binary scale to be “Art” or “not Art.” With the sole exception of the “Not art control,” when an object was classified as art, it was also most often rated above the half-point mark on the 100-point scale. These results suggest that both scales were largely measuring the same underlying construct. In addition, as can also be seen in Figure 2, which does show some fluctuation between object types, the 100-point scores potentially reveal both classification and participant confidence in their assessments, while the percentages most probably are a more direct measure of spontaneous classification itself.

As displayed in Table 1, even when considering typical art categories (i.e., Abstract and the Renaissance/Baroque paintings), there was a rather large range and standard deviation in what participants considered art between the individual examples. For example, abstract paintings ranged from being classified as art 100% of the time by a few (16.8%) participants to only being found to be art in one case (of 30 possible) by another. Looking to the individual participants, we found that only five individuals (4.4%) answered “yes” to Art classification for every trial across all types; even for the Renaissance paintings the percentage of participants classifying all of the images as art was only 72.6%. All other art types had participants answering “yes” 100% of the time in less than 20% of cases. Together these results suggest that participants often did not determine an object as art, even if it came from a category considered as producing “obvious” art examples, with assessments largely determined by the specific object. The range of classifications, especially the controls which showed rates at the high and low ends, further suggests that participants were able to routinely follow the task, and that the classification decisions were most probably not driven by the screen setting (e.g., appearing as digital reproductions).

### Art/not art classification and liking ratings

Moving to liking assessments of the objects, as shown in Table 1, the highest liking scores (combining objects classified as both art and not art) were found for the Renaissance/Baroque paintings (*M* = 39.0 on the 100-point scale). The lowest scores were found for the photos of everyday objects (*M* = 16.5). All other object types ranged from ~25 to 30. Significant correlations were also found between the above percentage scores for positive art classification, within each art type, and the mean liking ratings for the same categories ^τ^B range = 0.18–0.38, all *p*s < 0.02). As with the classification results above, the liking ratings of the object types also showed significant correlations between each category ^τ^B range = 0.31–0.58, all *p*s < 0.001).

**Figure 3:**
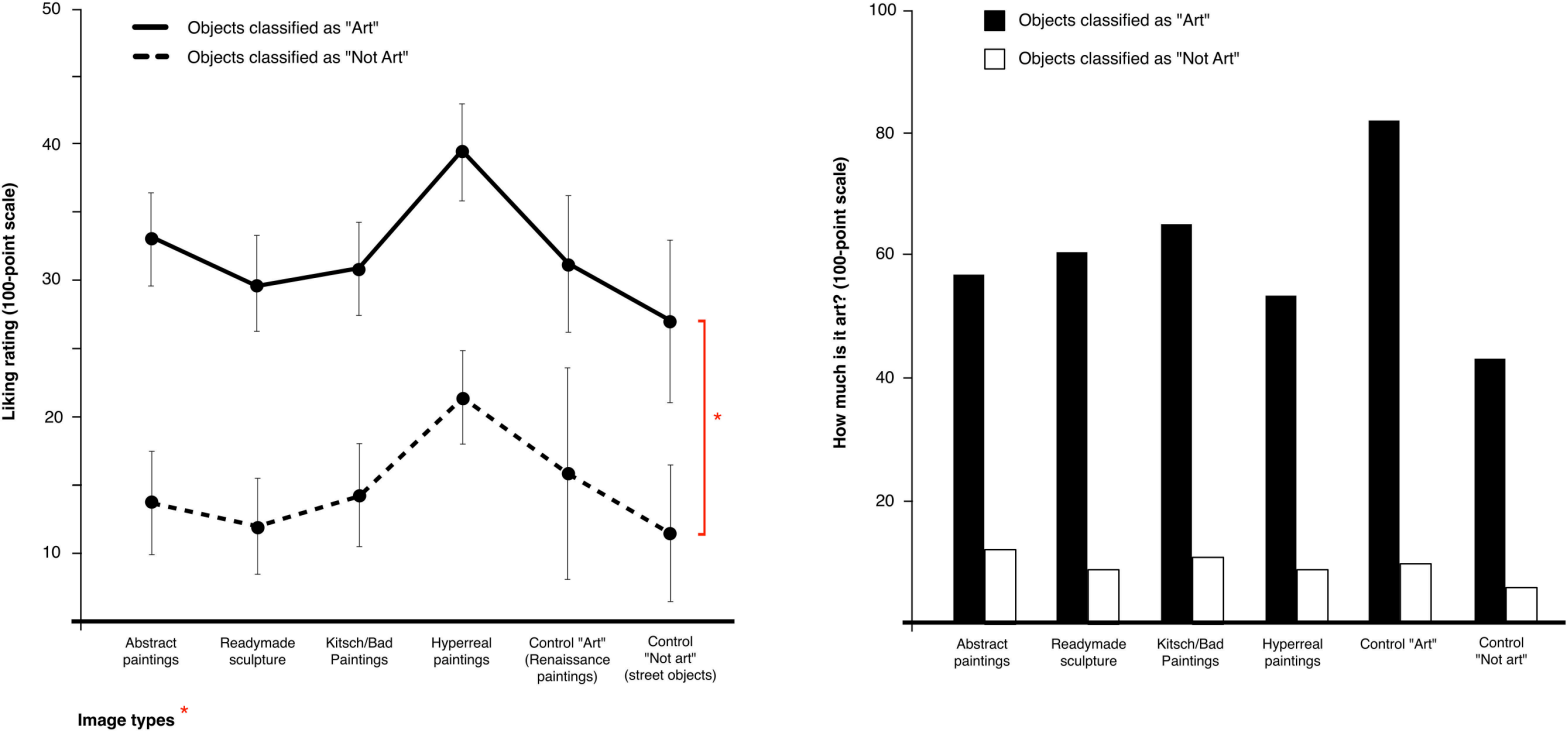
Difference in liking rating of image types based on classification as “Art” or “Not Art.” (^*^Repeated measures ANOVA: Arthood (yes/no) × category type (Abstract, Readymade, Hyperreal, Kitsch/Bad, Renaissance, Photos of everyday objects): significant main effect for Arthood *F*_(1)_ = 33.42, *p* < 0.001, pη^2^ = 0.71, and for category type, *F*_(5)_ = 3.46, *p* = 0.008, pη^2^ = 0.20).

As shown in Figure 3, critically, there was also a notable difference in liking ratings depending on whether an image was classified as art. This difference appeared to be consistent across all object types, with an overall Mean liking rating of objects classified as “art” of 35.5 vs. 14.6 for “not art,” resulting in a difference of 20.9 points (roughly 59%). To assess this difference, a repeated measures ANOVA was conducted with the factors *Arthood* (yes/no) × *Category type* (Abstract, Readymade, Hyperreal, Kitsch/Bad, Renaissance, Everyday photos). The ANOVA showed a main effect for Arthood [*F*_(1, 14)_ = 33.42, *p* < 0.001, $\eta_{p}^{2}$ = 0.71] and for Category [*F Greenhouse-Geisser* (2.91, 40.66) = 3.45, *p* = 0.02, $\eta_{p}^{2}$ = 0.20, Mauchly's test $\chi_{\left(14\right)}^{2}$ = 27.02, *p* = 0.02]. The interaction between Arthood x Category type was not significant [*F*_(5, 70)_ = 0.15, *p* = 0.98, Mauchly's test $\chi_{\left(14\right)}^{2}$ = 11.85, *p* = 0.63]. The partial eta squared measure also suggested that the Arthood status of the objects was associated with 71% of rating variance (including unexplained variance).

### What features of the object or processing experience contributed to classifying art?

Moving to the third main question, we then assessed which object features and processing experiences participants considered relevant when making the Art/Not-art classification tasks. To analyze this we looked to the individual items from the post-test questionnaire in which participants were asked to consider their classifications of the various images, and to rate the importance of each item when making this determination. These are first reported as individual correlation analyses (Kendall Tau b) in Table 2 (see also Supplementary Materials for Pearson product-moment results), comparing importance of the factors when making assessments with the percentage score for finding the entire set of objects to be art. We also report all correlations for both the composite of all art types and for individual varieties. However, we will mainly focus on the composite scores for the following discussion.

**Table 2:** Factors used in making art/not art decisions: correlation between 1 and 7 answer to “how important was the following factor in making your art/not art decisions” with Percentage of objects classified as art.

	**M**	**SD**	**Correlations**						
			**All (non-controls)**	**Abstract paintings**	**Readymade sculpture**	**Hyperreal paintings**	**Kitsch/bad paintings**	**Renaissance/baroque painting control**	**Control everyday objects**
Beauty	4.11	2.01	−0.153^*^	−0.186^*^	−0.115	0.019	−0.209^**^	0.067	0.023
Technical quality	4.69	1.71	−0.138	−0.150^*^	−0.138	−0.005	−0.145^*^	0.018	−0.059
Evidence of making	4.77	1.94	−0.088	−0.111	−0.090	−0.124	0.018	0.128	−0.131
Content	4.60	1.86	−0.073	−0.079	−0.021	0.020	−0.079	0.127	−0.007
Artwork style	5.53	1.50	−0.163^*^	−0.140	−0.189^**^	−0.110	−0.118	0.120	−0.078
Composition	5.15	1.60	0.024	−0.065	0.064	0.148^*^	−0.062	0.191^*^	0.080
Form	3.61	1.71	0.084	0.071	0.106	0.064	0.014	0.098	0.093
Colors or contrast	4.01	1.71	0.103	0.059	0.107	0.107	0.043	0.139	0.205^**^
Materials	3.69	1.72	−0.016	−0.078	0.020	−0.011	−0.017	0.157^*^	0.066
Expensive looking	2.38	1.62	−0.076	−0.125	−0.031	−0.034	−0.128	0.060	0.006
Evokes nostalgia	3.23	1.77	0.023	0.032	0.033	0.003	0.044	0.087	0.102
Challenges me	3.94	1.93	0.125	0.125	0.121	0.022	0.085	−0.012	0.093
Makes me uncomfortable	2.41	1.63	0.193^**^	0.171	0.196^**^	0.108	0.048	0.041	0.151^*^
Makes me safe, comfortable	3.16	1.85	0.047	0.015	0.018	0.039	0.064	−0.017	0.169^*^
Novelty	3.94	1.88	0.079	0.112	0.098	0.052	0.030	−0.037	0.116
Aligns with beliefs, values	2.78	1.99	−0.001	0.036	−0.006	−0.032	0.003	−0.115	0.065
Emotionally evocative	4.95	1.85	0.090	0.075	0.085	0.137	0.007	−0.012	0.142^*^
Thought-provoking	5.06	1.85	0.136	0.151^*^	0.127	0.129	−0.013	0.062	0.088
Felt a deeper meaning	4.59	1.84	0.071	0.057	0.086	0.064	0.028	0.104	0.041
Had no meaning, purpose	2.16	1.70	0.119	0.114	0.137	−0.022	0.174^*^	0.112	0.188^**^
I thought of experts' opinion	2.63	1.79	−0.135	−0.104	−0.094	−0.177^*^	−0.094	0.109	0.084
Made me see the world through artist's eye	3.73	1.88	0.127	0.099	0.156^*^	0.090	0.024	0.025	0.117
Every painting is automatically art	2.66	1.82	0.314^**^	0.269^**^	0.238^**^	0.164^*^	0.385^**^	0.107	0.216^**^

*Non-parametric Kendall Tau b*.

Results based on N = 114 (80 female, M age = 23.2, native German speaking) psychology students from the University of Vienna. Correlations are the result of individual Kendall Tau b analyses (two tailed). Correlations are uncorrected for multiple comparisons. Bold value indicates statistical significance at

* p < 0.05;

** *p < 0.01*.

The strongest correlation for classifying an object as art was found for the belief “Every painting is automatically art” suggesting a categorical strategy for addressing art classifications. At the same time, we also find a general pattern of answers which suggest a negative relation between reliance on traditional aspects of artistic appraisal and finding objects to be art. A negative correlation was found between relying on object *Beauty* and art classification, for both the composite average, as well as for the individual Abstract and Kitsch/Bad painting types. Similar results were also found for *Style* (composite score and Readymades) and *Technical Quality* (Abstract, Kitsch). These qualities, in conjunction with the above correlations, appeared to align more or less with features salient in the individual types of objects. For example, the beauty scores may have been more important in Abstract and Bad paintings because many individuals may consider them to not be beautiful and thus not art. Similarly, Abstract and Kitsch may have been considered to not be technically well-crafted and thus not art, in both cases suggesting either a categorical or/and a hedonic motive. On the other hand, positive correlations were found between considering objects to be uncomfortable-making, and “Art” classification in the composite and Readymade cases. This may suggest that those who were able to appreciate the conceptual or contextual importance of the objects found them to be art. A similar correlation was found for Readymades and being made to “see the world through the eyes of the artist.”

To identify potential patterns in use of these factors, we conducted a Principle Component Analysis on all 23 items (Varimax Rotation with Kaiser normalization, missing values replaced with group means). The Kaiser-Meyer-Olkin measure verified good sampling adequacy (KMO = 0.75; see Field, 2009). Bartlett's test of sphericity $X_{\left(253\right)}^{2}$ = 713.4, *p* < 0.001, also indicated sufficient correlations between items. Parallel analysis of the results (Monte Carlo Simulation, 1,000 permutated datasets; Henson and Roberts, 2006), returned seven significant components, explaining 65.01% of variance. As shown in Table 3, these included: (1) items involving the processing experience or impact on the viewer (thought provoking, challenge, evoked emotion); (2) pictorial qualities of the art body, (3) “comforting” qualities of beauty, positive emotions, and opinion-congruent values; (4) stylistic aspects; (6) social features, and (7) nostalgia. Last, and most notably, as also seen in the subsequent regression below, the 5th factor returned a collection of responses that appeared to combine categorical factors (belief that “every painting is automatically art”) with hedonic aspects (focus on “beauty” and “technical quality”). Note that these had also been significant in the above correlations, and here showed opposite loadings on this component between the categorical (i.e., positive loading) and hedonic (i.e., negative) factors, suggesting that participants relied on one or the other strategy, but not both in tandem.

**Table 3:** Principle component analysis of strategies used in determining art/not art classification.

	**Component**						
	**1 (processing experience)**	**2 (art body)**	**3 (comfort)**	**4 (style)**	**5 (categorical vs. hedonic)**	**6 (social)**	**7 (nostalgia)**
Eigenvalues	3.24	2.38	2.33	2.07	1.95	1.76	1.23
% explained variance	14.08	10.35	10.12	8.99	8.49	7.64	5.36
Multiple regression Prediction of “Art”	*B* = 3.22, *t* = 1.95, *p* = 0.05	*B* = 2.25, *t* = 1.36, *p* = 0.18	*B* = 0.81, *t* = 0.49, *p* = 0.63	*B* = −3.65, *t* = −2.22, *p* = 0.03	*B* = 6.02, *t* = 3.66, *p* < 0.001	*B* = −3.01, *t* = −1.83, *p* = 0.07	*B* = 2.25, *t* = 1.37, *p* = 0.18
**ITEMS^a^**							
Thought-provoking	0.803						
Emotionally evocative	0.691		0.356				
Challenges me	0.680						
Felt a deeper meaning	0.672						
See world through artist eye	0.656						
Form		0.824					
Colors or contrast		0.771					
Composition		0.590		0.435			
Materials		0.537					0.403
Novelty		0.464					−0.372
Makes me feel safe			0.819				
Makes me uncomfortable^*^			0.645				
Aligns with my beliefs, values			0.596				
Content			0.455	0.395			
Beauty(^*^)			0.374		−0.356		
Style(^*^)				0.738			
Evidence of making				0.731			
Every painting is art^*^					0.761		
Technical quality					−0.660		
Had no meaning, purpose					0.563	0.457	
Thought of expert' opinion						0.738	
Expensive looking						0.718	
Evokes nostalgia							0.627

*Results of Principle Component Analysis on all 23 items shown with Varimax Rotation (Kaiser Normalization, missing values replaced by group mean, 14 iterations). Total number of components (7) selected following Parallel Analysis (Monte Carlo simulation, 1,000 iterations). Total variance explained = 65.01%. Kaiser-Meyer-Olkin measure verified good sampling adequacy (KMO = 0.75; see Field, 2009). Bartlett's test of sphericity $X_{\left(\text{253}\right)}^{2}$ = 713.4, p < 0.001 indicated sufficient correlations between items. Results based on N = 114 (80 female, M age = 23.2, native German speaking) psychology students from the University of Vienna*.

* *Indicates significant correlation, individual Kendall Tau b analyses, between factor and classifying objects as art (% Yes)*.

(*) *Indicates negative correlation*.

a *Items are verbatim terms given to participants in conjunction with the question, “how important was the following factor in making your determination of art or not art?” Item loadings below 0.35 on specific components not shown*.

To further analyze the connection of these components with the art classifications, factor scores were then entered in a multiple regression with art classification as the dependent variable (forced entry method). In this case, note that, critically, the dependent factor was shown to be normally distributed via Shapiro-Wilk (*p* = 0.22). This is reported in Table 3 (top), and showed significance for Processing (*p* = 0.05), Artistic style (*p* = 0.03), and, most significantly, Categorical vs. hedonic components (*p* < 0.001) as predictors of art classification. Similar regression analysis with liking as the dependent variable showed that again the categorical vs. hedonic component (*B* = 2.63, *p* = 0.02) and pictorial qualities (*B* = 3.09, *p* = 0.01) were the only significant predictors.

**Figure 4:**
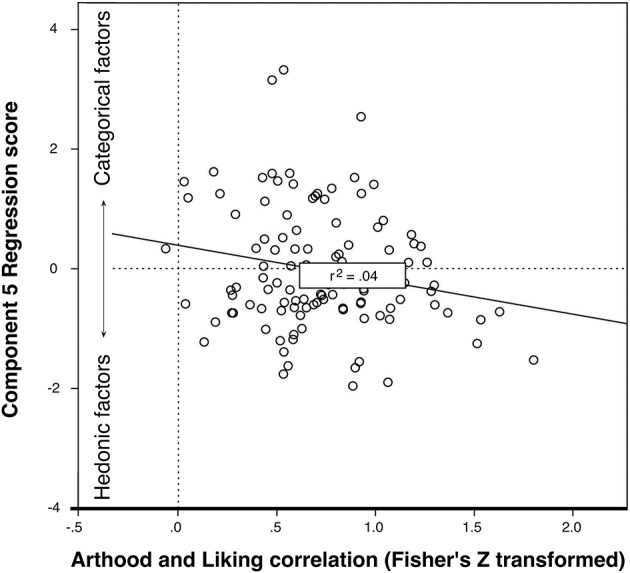
Relation between object appraisal and liking correlation (bottom) and use of Hedonic vs. Categorical strategies for classification as “Art” or “Not Art”.

Finally, to assess whether the use of categorical vs. hedonic strategies (again as reflected in the calculated score for Component 5) might reveal different degrees of relation between Liking and art classification, we calculated a correlation score between both 100-point measures over all artworks for each participant. Linear regression analysis of the correlations (with Fisher's Z transformation to normalize the distribution) and the score for the Categorical vs. hedonic component was significant [*F*_(1, 113)_ = 3.51, *r* = –0.201, *p* = 0.03]. As can be seen in Figure 4, participants with scores reflecting use of more categorical factors showed a lower correlation between liking and art classification than those using hedonic factors.

### Who did and did not classify objects as art? expectations and personality

Last, we assessed our fourth question regarding interpersonal differences in personality, tastes, expectations, art experiences and the resulting assessment- of objects as art. As in the section above, this was assessed by conducting Kendall Tau b correlation analyses between individual items on the post-test questionnaires and the percentage scores for classifying objects as art. Results are reported separately for all individual scales and art types in Tables 4–**9** (see also Supplementary Materials for Pearson coefficients). We briefly describe some notable findings in the following subsections, relating this to the previous literature on art classifications. This is then again followed by a final data reduction (PCA) and final multiple regression below.

**Table 4:** Correlation with classifying objects as art: art training, education and involvement.

	**All (non-control) (% art)**	**Abstract**	**Ready-made**	**Hyper-real**	**Kitsch/bad painting**	**Control: renaissance/baroque painting**	**Control: everyday objects**	**Liking (all art)**
**Art training and education (Chatterjee et al., 2010)**								
Number of studio art classes (H.S.+)	0.137	0.170^*^	0.103	0.139	0.050	0.006	0.034	0.093
Number of art history classes (H.S.+)	0.153^*^	0.255^*^	0.116	0.084	0.112	0.089	0.110	0.039
Number of art theory or aesthetics classes (H.S.+)	0.125	0.183^*^	0.103	0.030	0.090	0.085	0.030	−0.081
Hours spent making visual art	0.135	0.100	0.069	0.159^*^	0.125	−0.016	**0.143**	0.007
**Objective art involvement (Leder et al., 2014)**								
How often visit art museums?	0.211^**^	0.215^**^	0.221^**^	0.155^*^	0.104	0.043	0.121	−0.022
How often read art books?	0.234^**^	0.288^**^	0.208^**^	0.151^*^	0.154^*^	−0.078	0.089	0.010
How often look at pictures of art?	0.311^**^	0.315^**^	0.283^**^	0.231^**^	0.184^**^	0.035	0.103	0.035
How often visit art events (lectures, etc.)	0.211^**^	0.240^**^	0.190^**^	**0.130**	0.203^**^	0.000	0.187^**^	0.058
**Art knowledge and comfort (Pelowski**, **2015****)**								
I am comfortable looking at and discussing art.	0.279^**^	0.349^**^	0.258^**^	0.143^*^	0.141^*^	0.115	0.037	0.049
I am knowledgeable about art	0.299^**^	0.333^**^	0.270^**^	0.198^**^	0.199^**^	0.025	0.111	0.059
Art is important	0.300^**^	0.290^**^	0.268^**^	0.208^**^	0.149^*^	0.079	0.110	0.052
I enjoy being challenged by art	0.316^**^	0.340^**^	0.296^**^	0.204^**^	0.149^**^	0.079	0.133	0.053
I am interested in art	0.337^**^	0.376^**^	0.287^**^	0.214^**^	0.189^*^	0.124	0.117	0.051

*Non-parametric Kendall Tau b*.

Results based on N = 114 (80 female, M age = 23.2, native German speaking) psychology students from the University of Vienna. Correlations are the result of individual Kendall Tau b analyses (two tailed). Correlations are uncorrected for multiple comparisons. Bold value indicates statistical significance at

* p < 0.05;

** *p < 0.01*.

#### Art training and general expectations or beliefs about art

We find a pattern of responses indicative of an individual with an interest in and open enjoyment of art, and who also shows a wide interest in more complex art-induced reactions and more contemporary art examples. First, in regards to art training, education, and attitudes about art (Table 4), significant, positive correlations were identified with objective aspects of art exposure (i.e., number of museum visits, propensity to read books about art, from the Leder et al., 2014 items) and with personal factors related to self-assessed belief that one was knowledgeable about art, interested in art, comfortable looking at and discussing art, and that one enjoyed being challenged by art and that art itself was important. In most cases, significance was also found for all main object categories. Also of note, no items among the above scales significantly correlated with liking. In regards to participants' expectations about art's desired qualities and impact (Table 5), the belief that art examples should evoke “surprise,” “disruptions” and “uncomfortable feelings” and “transformation” or personal change were associated positively with art classifications. Again these were also not significantly correlated with liking ratings. On the other hand, belief that “realism leads to better art,” or that good art should evoke “pleasure” or “tranquility” showed a significant negative correlation with art classification.

**Table 5:** Correlation with classifying objects as art: general Artwork beliefs and preferences.

	**All (non-control) (% art)**	**Abstract**	**Ready-made**	**Hyper-real**	**Kitsch/bad painting**	**Control: renaissance/baroque painting**	**Control: everyday objects**	**Liking (all art)**
The best art is difficult or challenging	0.021	0.010	0.002	0.066	0.047	0.034	0.157^**^	0.008
The best art makes you feel	0.056	0.033	0.043	0.052	0.091	0.078	0.076	0.178^*^
The best art makes you think	0.064	0.046	0.057	0.119	−0.017	0.036	0.098	0.033
The best art primarily is pleasurable	−0.180^**^	−0.220^**^	−0.168^*^	−0.071	−0.104	−0.070	−0.080	0.005
The best art should make you feel tranquil or harmony	−0.196^**^	−0.223^**^	−0.187^*^	−0.096	−0.082	−0.064	−0.108	−0.009
The best art should make you feel insight	−0.074	−0.076	−0.028	−0.064	−0.114	0.083	0.014	−0.055
The best art should make you feel Catharsis or relief	−0.039	−0.058	0.001	−0.014	−0.049	−0.035	0.034	0.015
The best art should make you feel transformation or personal change	0.140^*^	0.097	0.135	0.129	0.053	0.048	0.132	0.086
The best art should make you feel disrupted or uncomfortable	0.166^*^	0.081	0.197^*^	0.124	0.016	0.001	0.162^*^	**0.130**
The best art should make you feel surprise	0.148^*^	0.100	0.188^**^	0.131	0.049	0.008	0.010	**0.131**
The best art should make you feel curiosity	0.086	0.067	0.100	0.150^*^	−0.031	0.083	0.051	0.100
The best art should make you feel a sense of novelty	0.039	0.036	0.077	0.029	−0.018	0.086	0.010	0.073
The more realistic the painting, the better the artist	−0.209^**^	−0.279^**^	−0.214^**^	−0.029	−0.194^*^	0.043	−0.069	−0.101
Anybody could produce abstract art	−0.048	−0.142^*^	−0.093	−0.002	0.049	−0.007	0.080	0.028
Everyone who can draw something realistically is a good artist	0.011	−0.041	−0.005	0.036	0.085	0.174^*^	0.046	0.087

*Non-parametric Kendall Tau b*.

Results based on N = 114 (80 female, M age = 23.2, native German speaking) psychology students from the University of Vienna. Correlations are the result of individual Kendall Tau b analyses (two tailed). Correlations are uncorrected for multiple comparisons. Bold value indicates statistical significance at

* p < 0.05;

** *p < 0.01*.

By way of reference, in the study by Tröndle et al. (2014) participants who classified their example as art also reported being more surprised, made to think, and moved emotionally. These factors might also be linked to rather traditional or art-naive expectations about art's quality, referring to pre-Modern examples. Looking to the breakdown between object types, we do find that significant correlations were often found for Readymade and Abstract art, whereas they were not as strong for Kitsch and Hyperrealistic (i.e., mimetic) examples.

#### Art types preference

As shown in Table 6, which lists preference for art types, we find significant correlation between preferring more modern art forms (Abstract, Readymade, Avant Garde, Cubism, Surrealism, Pop art, Graffiti, Conceptual art) and art classifications in general, as well as with Abstract art examples (all cases) and with Readymades and Hyperreal paintings for several cases. On the other hand, preference for more traditional art forms (Classic, Kitsch, Representational, Impressionistic) showed low correlation with art classifications in general or within the specific object types.

**Table 6:** Correlation with classifying objects as art: art-type preference.

	**All (non-control) (% art)**	**Abstract**	**Ready-made**	**Hyper-real**	**Kitsch/bad painting/drawing**	**Control: renaissance/ baroque painting**	**Control: everyday objects**	**Liking (all art)**
Abstract	0.222^**^	0.265^**^	0.207^**^	0.153^*^	0.051	0.033	0.110	−0.006
Readymade	0.223^**^	0.234^**^	0.254^**^	0.100	0.081	0.100	0.082	0.188^*^
Classic	−0.068	−0.056	−0.060	−0.040	0.039	0.133	−0.087	−0.080
Kitsch	0.037	0.062	0.000	−0.035	0.130	0.067	0.050	0.056
Avant Garde	0.388^**^	0.378^**^	0.369^**^	0.304^**^	0.172^*^	0.087	0.203^*^	0.153^**^
Representational	0.143^*^	0.149^*^	0.086	0.132	0.074	0.001	−0.031	0.049
Fantasy	0.056	0.022	0.001	0.041	0.144^*^	−0.007	−0.017	0.107
Graffiti	0.173^**^	0.167^*^	0.106	0.127	0.178^**^	−0.007	0.152^*^	0.176^*^
Digital art	0.166^*^	0.120	0.105	0.200^**^	0.157^*^	0.030	0.085	0.189^**^
Impressionism	0.031	0.077	0.008	0.046	0.070	0.088	−0.022	0.028
Cubism	0.162^*^	0.184^*^	0.115	0.142	0.084	0.148^*^	0.055	−0.070
Surrealism	0.236^**^	0.251^**^	0.222^**^	0.136	0.147^*^	0.136	0.009	0.115
Pop art	0.193^**^	0.255^**^	0.169^*^	0.100	0.101	−0.040	−0.040	0.155^*^
Conceptual art	0.184^*^	0.221^**^	0.227^**^	0.066	0.060	0.058	−0.031	−0.011

*Non-parametric Kendall Tau b*.

Results based on N = 114 (80 female, M age = 23.2, native German speaking) psychology students from the University of Vienna. Correlations are the result of individual Kendall Tau b analyses (two tailed). Correlations are uncorrected for multiple comparisons. Bold value indicates statistical significance at

* p < 0.05;

** *p < 0.01*.

#### Expectations for visiting art/museums

Looking to the stated expectations for viewing art or visiting art museums (from Tröndle et al., 2014; Table 7), we find a significant positive correlation with desire to “experience a deep connection to art,” as well as to “be surprised.” This was found for both the composite and Abstract and Readymade cases. Positive correlations were also found, however only in the case of Abstract art, with a desire to “improve understanding of the fine arts” and to “see familiar artworks,” possibly again suggesting a tie with expertise. On the other hand, negative correlation was found between the composite, as well as the Abstract type, and a desire to see “famous artworks.” For comparison, the earlier Tröndle et al. study found significance with “seeing famous artworks,” as well as a desire to “have thoughts provoked” and to be “part of an exhibition with all the senses” (direction of relations not reported). However, the earlier study was only conducted with one art example which itself was of a quite unique variety (installation consisting of commentary written on the museum walls). Presumably, the present findings may coincide with a more general propensity to classify objects as art.

**Table 7:** Correlation with classifying art: expectations for visiting art/museum (from Tröndle et al., 2014).

	**All (non-control) (% art)**	**Abstract**	**Ready-made**	**Hyper-real**	**Kitsch/bad**	**Control: renaissance/baroque painting**	**Control: everyday objects**	**Liking (all art)**
Have my thoughts provoked	0.116	0.127	0.145^*^	0.122	0.003	0.185	0.098	0.110
Art design to be convincing	0.008	0.066	0.011	0.070	−0.029	−0.066	−0.044	0.067
Enjoy silence of museum space	0.027	0.085	0.022	−0.023	0.011	0.098	−0.009	0.035
Improve understanding of arts	0.115	0.171^*^	0.108	0.024	0.057	0.144	0.077	0.007
Have a nice time with family/friends	−0.083	−0.058	−0.077	−0.057	−0.046	0.062	−0.083	−0.032
Be part of the art exhibitions with all my senses	0.104	0.123	0.099	0.085	0.009	0.048	0.036	0.050
Experience deep connection to art	0.186^*^	0.234^**^	0.161^*^	0.087	0.123	0.028	0.083	0.092
See something familiar which I already know	0.087	0.150^*^	0.080	0.024	0.064	0.070	0.023	−0.001
Experience the beauty of artworks	−0.045	−0.016	−0.023	−0.007	0.021	0.024	−0.010	−0.029
Be entertained	−0.036	−0.019	−0.038	−0.038	−0.012	0.054	−0.023	−0.108
Be surprised	0.138^*^	0.154^*^	0.186^**^	0.107	0.013	0.112	0.044	0.013
See famous artworks	−0.183^*^	−0.153^*^	−0.118	−0.134	−0.114	0.242^**^	−0.072	−0.174^*^

*Kendall Tau b*.

Results based on N = 114 (80 female, M age = 23.2, native German speaking) psychology students from the University of Vienna. Correlations are the result of individual Kendall Tau b analyses (two tailed). Correlations are uncorrected for multiple comparisons. Bold value indicates statistical significance at

* p < 0.05;

** *p < 0.01*.

#### Personality

Among general personality measures (Table 8), significant correlations were found with the Openness subset of the Big Five Inventory. This held true both for the all-art composite and for all art sub-types. This suggests a willingness to seek out unfamiliar or novel encounters, and has also been shown to arise in several studies with art, correlating with, among other findings, a deeper appreciation for and engagement with the arts and creativity (Kaufman, 2013; Myszkowski et al., 2014; Fayn et al., 2015; Kaufman et al., 2015). Conversely, the need for cognitive closure correlated negatively with art classifications in composite and all sub-types with the exception of Kitsch. This item suggests a disliking of ambiguity or desire for clear classifications, and has shown a negative correlation with Openness (Roets and Van Hiel, 2011), which was also found in the present study (^τ^B = −0.266, *p* < 0.001). Note also that no significant correlations were found for the personality measures and liking the art. This may suggest, once again, that these aspects of personality especially involve the willingness to consider “atypical” art types as artworks proper, and thus a category-related rather than hedonic assessment of art/not art.

**Table 8:** Correlation with classifying objects as art: personality measures.

	**All (non-control) (% art)**	**Abstract**	**Ready-made**	**Hyper-real**	**Kitsch/bad**	**Control: renaissance/baroque painting**	**Control: everyday objects**	**Liking (all art)**
BFI Extraversion	−0.128	−0.078	−0.134^*^	−0.088	−0.103	−0.076	−0.203^*^	−0.099
BFI Agreeableness	−0.063	−0.065	−0.072	0.008	−0.002	−0.062	−0.089	0.037
BFI Conscientiousness	−0.065	−0.066	−0.051	0.020	−0.051	0.182^*^	−0.009	0.048
BFI Neuroticism	0.020	0.017	0.055	0.008	0.035	0.086	0.007	0.009
BFI Openness	0.284^**^	0.311^**^	0.239^**^	0.199^**^	0.141^*^	0.036	0.057	0.091
Need for Cog. Closure	**−0.112**	−0.091^*^	−0.133^*^	−0.132^*^	0.034	0.046	−0.066	−0.002
Creative personality	0.094	0.078	0.051	0.124	0.031	0.045	0.137	0.054

*Kendall Tau b*.

Results based on N = 114 (80 female, M age = 23.2, native German speaking) psychology students from the University of Vienna. Correlations are the result of individual Kendall Tau b analyses (two tailed). Correlations are uncorrected for multiple comparisons. Bold value indicates statistical significance at

* p < 0.05;

** *p < 0.01*.

#### Sociological breakdown of participant activities and tastes

Viewer tastes and activities, following the social profile questions from Hanquinet (2013), are shown in Table 9. Significant correlations were found for liking electronic/dance music and for not enjoying pop music, as well as for liking essays and art books. Positive correlation was also found with the creative activities of photography and making paintings or drawings, as well as with highbrow activities related specifically to art—visiting commercial galleries, art centers, and museum. Note also that we did not find correlation with other, perhaps more classical, types of fine art such as ballet and theater. Significance was also found for having purchased an art book, while no significant correlations were found with more lowbrow forms of leisure. This collection of factors, in addition to our participants' age and the fact that they were college students, would appear to suggest the “omnivorous” art viewer noted by Hanquinet (2013): An individual who is educated, younger (below age 35), and who often visits and seeks out art, but who, unlike past findings with art lovers, is not necessarily exclusively tied to enjoyment of generally “high culture” activities (opera, classical music), rather such a person has a blend of low and high Avant-garde tastes.

**Table 9:** Correlation with classifying objects as art: Hanquinet (2013) Social profile of tastes and interests.

	**All (non-control) (% art)**	**Abstract**	**Ready-made**	**Hyper-real**	**Kitsch/bad**	**Control: renaissance/baroque painting**	**Control: everyday objects**	**Liking (all art)**
**TASTE IN MUSIC**								
Prefer opera, classical music	0.008	0.007	0.045	0.055	−0.068	−0.044	−0.062	−0.054
Jazz	0.095	0.109	0.073	0.074	0.067	0.095	−0.031	−0.056
Electronic, dance	0.139^*^	0.181^**^	0.139^*^	0.114	0.074	−0.017	−0.018	0.174^*^
Hard rock	0.082	0.100	0.059	0.061	0.144^*^	0.115	0.023	0.102
Pop	−0.162^*^	−0.147^*^	−0.205^**^	−0.103	−0.008	0.009	−0.082	0.040
World music	0.019	0.076	0.001	−0.004	0.061	0.072	−0.009	−0.003
Folk	0.037	.026	0.066	−0.013	0.102	0.091	0.086	0.074
Schlager	−0.014	−0.043	−0.067	0.044	0.068	−0.137	0.145	0.049
**TASTE IN BOOKS**								
Prefer reading practical books (e.g., cooking)	−0.018	0.013	−0.033	0.019	−0.034	−0.028	−0.061	−0.008
Detective novels, comics	−0.045	−0.080	−0.039	0.012	−0.016	0.057	0.049	−0.036
Classical literature	0.062	0.070	0.095	0.032	0.001	0.148	−0.004	−0.048
History books, non-fiction	0.047	0.001	0.023	0.062	0.076	0.028	−0.036	0.035
Art books	0.218^**^	0.266^**^	0.182^*^	0.106	0.190^**^	−0.050	0.105	0.047
Essays	0.250^**^	0.240^**^	0.237^**^	0.194^**^	0.162^*^	−0.052	0.093	0.102
**HIGHBROW ACTIVITIES**								
I have gone to theater	−0.011	−0.029	0.026	0.076	−0.121	−0.154	0.001	0.099
Concerts of classical music or jazz	0.078	0.145	0.072	0.013	0.091	−0.032	−0.068	−0.062
Dance performance	−0.019	−0.006	−0.053	−0.005	0.006	−0.028	−0.068	0.014
Opera	0.108	0.076	0.124	0.083	0.000	0.006	0.067	0.039
Commercial art galleries	0.220^**^	0.218^**^	0.183^**^	0.151^*^	0.119	−0.025	0.152	0.039
Contemporary art centers	0.190^**^	0.182^*^	0.199^**^	0.182^*^	0.053	−0.040	0.157	0.047
Museums, art exhibitions	0.162^*^	0.174^*^	0.157^*^	0.121	0.078	0.011	0.130	0.052
Ballet	0.045	0.005	−0.094	0.054	0.058	0.067	−0.004	0.079
**CREATIVE ACTIVITIES**								
I have participated in dance	0.104	0.122	0.048	0.061	0.151^*^	0.105	0.085	0.040
Theater	0.091	0.061	0.056	0.071	0.114	0.108	0.079	0.095
Photography	0.289^**^	0.200^*^	0.230^**^	0.274^**^	0.248^**^	0.186^*^	0.227^*^	0.143^*^
Painting/drawing	0.222^**^	0.153^*^	0.181^**^	0.186^**^	0.198^**^	−0.045	0.172^*^	0.053
Playing music	0.093	0.042	0.061	0.066	0.209^**^	0.021	0.132	0.103
Writing	0.100	0.074	0.074	0.044	0.074	0.056	0.055	0.047
**LEISURE ACTIVITIES**								
I have visited friends, family	0.022	0.020	0.015	0.070	−0.036	−0.033	0.053	0.044
Watched TV	−0.005	−0.034	0.006	0.092	−0.020	−0.030	0.144	0.082
Read a book	−0.014	−0.046	0.011	−0.025	−0.081	0.153	0.028	−0.015
Done odd jobs (e.g., gardening, fixing something in the house)	−0.059	0.019	−0.073	−0.069	0.018	0.122	0.007	−0.052
Gone out to eat (dinner)	0.096	0.174^*^	0.120	0.035	−0.027	−0.135	0.083	0.036
Played sports	−0.093	−0.061	−0.080	−0.047	−0.030	0.076	0.016	−0.099
Listened to the radio, music	−0.001	0.025	0.000	0.032	−0.095	0.134	−0.073	0.037
Gone to the cinema	−0.004	0.073	0.041	−0.061	0.005	−0.084	0.032	−0.058
Attended a sporting event	−0.006	−0.034	−0.048	−0.003	0.068	−0.072	0.117	0.054
Played a board or video game	0.098	0.030	0.085	0.090	0.111	0.014	0.148	−0.020
**PURCHASE OF ART**								
Have purchased genuine art	0.117	0.124	0.089	0.104	0.085	−0.066	0.005	0.042
Purchased an art reproduction	0.143	0.188^*^	0.090	0.113	0.088	−0.114	−0.022	−0.022
Purchased an art book	0.216^**^	0.203^*^	0.242^**^	0.178^*^	0.097	−0.081	0.139	0.045

*Kendall Tau b*.

Results based on N = 114 (80 female, M age = 23.2, native German speaking) psychology students from the University of Vienna. Correlations are the result of individual Kendall Tau b analyses (two tailed). Correlations are uncorrected for multiple comparisons. Bold value indicates statistical significance at

* p < 0.05;

** *p < 0.01*.

#### Principle component analysis of interpersonal factors, and relation with classification

Finally, as was done with the classification strategies above, a Principle Component Analysis was conducted on the personality/taste-related items in order to allow for a better picture of the general participant types, and what factors may be indicators of propensity to classify objects as art. The analysis combined all 114 measures reported in the section above (Tables 4–9). Parallel analysis (Monte Carlo simulation, 1000 permutated datasets) suggested five components. A final model (Maximum Likelihood Estimation, Direct Oblimen rotation with Kaiser normalization, missing values replaced by group Mean, 75 iterations, KMO = 0.30; Bartlett's $X_{\left(5,050\right)}^{2}$ = 5,462.9, *p* < 0.001), explained 33.69% of variance. Here we employed a Direct Oblimen rotation because this allows for either an oblique or an orthagonal solution. We expected that certain items would be reported by individuals in differing outcomes, thus this rotation was expected to provide a more natural fit for the data. It should also be noted that the Kaiser-Meyer-Olkin measure (above) did report a rather lowish sampling adequacy, due to the high number of included items.

Components and main loading items are shown in Table 10, and constituted: (1) highly *art interested* individuals, combining mostly art attitudes that art is important, that they are interested in art, enjoy art challenge, are comfortable discussing art and consider themselves knowledgeable; as well as Openness to Experience; (2) factors that can be considered to identify *experience interested* individuals, although not displaying specific interest or knowledge about art *per se*. (3) *Entertainment seeking* individuals, with for example expectations of going to museums in order to be entertained, to see famous artworks, have time with family, and who were not seeking a challenge when viewing art. (4) Individuals with rather *classic taste* (interested in opera, jazz, impressionist painting, and not in newer forms of art or music such as electronica), and (5) individuals who were not interested in, or an active patron of the arts (*art avoider*).

**Table 10:** Main factors resulting from principle component analysis of personality measures.

	**1 (Art interested)**	**2 (experience seeker)**	**3 (entertainment seeker)**	**4 (classic taste)**	**5 (art avoider)**
Eigenvalues	15.13	6.00	4.79	4.19	3.92
% explained variance	14.98%	5.94%	4.74%	4.15%	3.88%
Multiple Regression Prediction of “art” classification	B = 9.25, *t*_(107)_ = 5.71, *p* < 0.001	B = −0.02, *t*_(107)_ = −0.01, *p* = 0.99	B = −0.58, *t*_(107)_ = −0.36, *p* = 0.72	B = −3.00, *t*_(107)_ = −1.87, *p* = 0.06	B = 0.09, *t*_(107)_ = 0.58, *p* = 0.57
**ITEMS^a^**					
Art expectations: Art is important	0.787				
Art expectations: interested in art	0.615				
Art expectations: enjoy challenge	0.567				
Art expectations: comfortable looking, discuss art	0.566				
Art expectations: I am knowledgeable about art	0.333				
Museum expectation: experience deep connection	0.487	0.288			
Museum expectation: see something familiar	0.332				
Museum expectation: art design to be convincing		0.769			
Museum expectation: thought-provoking		0.649			
Museum expectation: be entertained			0.850		
Museum expectation: see famous artworks			0.415		
Museum expectations: improve art understanding	0.361				
Best art: more realistic painting, better artist	−0.360				
Best art: makes you think		0.581			
Best art: makes you feel		0.569			
Best art: make you feel disrupted, uncomfortable			−0.285		
BFI Openness	0.545				
BFI Extravert			0.333		
Hanquinet taste music: opera or classical				0.789	
Hanquinet taste music: jazz				0.663	
Hanquinet taste music: electronic or dance				−0.405	
Hanquinet taste art: impressionism				0.322	
Hanquinet culture activities: opera					−0.655
Hanquinet culture activities: ballet					−0.636
Hanquinet culture activities: theater					−0.614
Hanquinet culture activities: art museum					−0.327
Hanquinet leisure activities: visited friends/family		0.308			

*Results of Principle Component Analysis on 114 items (Tables 4–9) with Direct Oblimin Rotation (Kaiser Normalization, missing values replaced by group Mean, 75 iterations, KMO = 0.30; Bartlett's $X_{\left(5,050\right)}^{2}$ = 5,462.9, p < 0.001). Total number of components (5) selected following Parallel Analysis (Monte Carlo simulation, 1,000 iterations). Total variance explained = 33.69%. Results based on N = 114 (80 female, M age = 23.2, native German speaking) psychology students from the University of Vienna*.

a *Items loading below 0.3 on specific components omitted*.

This breakdown of participants appeared to roughly coincide with the general motivations for visiting art museums above, as well as Hanquinet's (2013) previous findings using similar scales. For example, this latter study identified six categories including: (1) “omnivorous,” educated, younger visitors who frequently visit and seek both high- and low-brow culture—potentially our experience seekers; (2) “art lovers” who also share omnivorous tastes and are highly interested in art; individuals with classical, conservative/traditional tastes, who have education and wealth, and seek out art or other cultural experiences (3,4); and (5) individuals who do not visit museums or do not seek out art.

In order to further consider the underlying question of how these individual types might predict propensity to classify objects as art, individual factor scores were computed for each factor for each participant and entered into a multiple regression (forced entry) with percentage of objects classified as art as the dependent variable. (Note that, despite the potential for multicolinearity with Oblimin rotated components, all Variance Inflation Factors were below). Results are reported at the top of Table 10. Only the first *Art interested* factor was a significant indicator of classification, constituting an increase in 9.25% points (unstandardized *B*) in the assessment of objects as art for each increase of 1 point on the component scale (intercept = 60.74%). The fourth, *classic taste*, group showed a non-significant trend of predicting *lower* classification of art (*p* = 0.06). Note also that the art avoider unequivocally did not show significance, suggesting in sum that classification propensity may be tied more to an interest in and an openness to differing art forms, rather than interest in culture or art itself.

Finally, analysis of correlations between the scores for the art interest components and the scores from the earlier PCA analysis of classification strategies showed a significant, positive relation for art interestedness with a focus on processing experience (Component 1 in Table 3 above, *r* = 0.27, *p* = 0.003) as well as with a focus on the art body (Component 2, *r* = 0.22, *p* = 0.01). Focus on processing experience was also found to be correlated with Experience seeking personalities (*r* = 0.52, *p* < 0.001). Interestingly, however, no personality correlated significantly, or to even a non-significant notable degree with the Hedonic vs. Categorical scale. In addition, although the art interest personality predicted propensity to classify objects as art, it did not predict propensity to like those objects: Correlation between personality component 1 (Art interest) and art liking, *r* = 0.122, *p* = 0.20. Regression analysis between the art interest component and the arthood/liking correlation scores [*F*_(1, 113)_ = 2.00, *p* = 0.16], as well as between arthood/liking correlation scores and the processing experience focus [Component 1 in Table 3; *F*_(1, 113)_ = 0.72, *p* = 0.40] were also not significant.

## General discussion and conclusion

The aim of this study was to consider the classification of images as works of art. This followed a line of argument, and a handful of recent empirical findings, that participants in empirical studies of “art”-viewing may not necessarily agree that the stimuli presented—even if chosen from the canon of established and respected artistic examples—are bona fide works of “art.” Thus, it was further argued, this difference in classification—although almost never directly assessed in previous studies—may play a role in, or even call into question, past empirical results regarding art-related responses or judgments. Here, artwork classification was therefore tested by systematically showing participants a number of types of images and asking for their spontaneous classifications as art/not art, as well as their appraisals for liking, with the goal of addressing the questions: (1) Do individuals automatically classify art images, presented in typical empirical art study, as works of art? (2) Does the art classification significantly relate to their aesthetic appreciation? (3) How does propensity toward art classification correlate with different appraisal strategies (e.g., hedonic, categorical), and (4) with other interpersonal factors?

Beginning with the first question: We do find evidence that viewers often *did not* classify objects as art. This in turn was highly dependent on the art type. Among our target categories, positive art classifications ranged from roughly 75% of Abstract and “Kitsch”/poorly executed paintings, to less than 50 percent of Readymade sculptures, 40% of Hypperrealistic paintings, and less than 15 percent of “Non art” photos of everyday objects. Whereas, participants classified 95% of Renaissance or Baroque pieces as art. Classification was further varied for almost all individuals within the different categories themselves. Individuals came to 100% classifications of all examples in a category (i.e., all examples of Abstract art) in less than 20% of cases. Even among our control Renaissance and Baroque paintings, only 72.6% of participants found every example to be art.

This finding in turn poses implications for past and future empirical art research. We support the suggestion that viewers—presented with a string of images, often subdivided between varieties, and asked to look, process, and/or to make ratings—may not necessarily, or automatically, believe what they are viewing is art. Even more, in answer to question two, art classification showed a strong, significant connection to preference. Liking of images classified as art was roughly 20 points higher (on the 100-point scale) than those found to be not art. This was also consistent between the art types (Figure 3), and between the individual viewers, accounting for roughly 71% of liking rating variance. While the present study cannot directly test causality, this does certainly call into question past, as well as future, studies which include appraisal or emotional response. For example, studies which show systematic differences in appraisal, reward, or engagement with certain art types as compared to others—e.g., the well-documented lay-viewer dislike of Abstract art—may be partially shadowed by their propensity to not view such paintings as artworks. Transversely, dislike for such examples may lead to lower arthood classifications.

Thus, we encourage researchers to consider this factor when composing future research. It may be pertinent to ask, either in follow-up or as a main aspect of a study, if individuals believe what they are viewing is art. Our evidence also suggests a systematic difference in classification between art varieties, tied most probably to more or less traditional styles, suggesting that researchers should especially be careful when using the latter variety, and/or employ post-study validation of art classifications. The more general connection between classification and appraisal, as well as order of such appraisals, is an important target for future systematic research.

Moving to our two additional questions, our study also provides evidence regarding who was, and why individuals were, making certain classifications, which might also be used as a base for future research. In our exploratory analyses, we find that the highest correlations to positive art classification was employing the belief that every painting or artist-made image is automatically a work of art. This suggests a categorical motive, as reviewed in the introduction. This argument is also supported by the comparatively lower rate of classification for Readymades and Hyper realistic paintings, with viewers potentially not seeing them as artist-made objects and thus not art examples. At the same time, we also found evidence for hedonic aspects—specifically the use of beauty, technical quality, and style as means of making classifications. However, these strategies were significant predictors for *not* classifying art. Interestingly, both hedonic factors and the categorical factor above, loaded onto the same general component in the PCA, suggesting that they are somewhat mutually exclusively employed by participants. Thus, while our findings tangentially coincide with Hagtvedt and Patrick's (2008) study, which also reported a complex mix of hedonic and categorical determinants, we can add the new argument here that it may be the selection of strategies that is a key element which should be considered in future assessment of participants or study results. At the same time, the complete absence of individuals who found no images to be art would also suggest against a “transferability thesis”-based motive (Currie, 1985). Happily, the fact that all participants did find at least some examples to be “art” suggests that—as is the implicit basis for most empirical study—they do indeed seem willing to treat images on a screen as an artwork, with their classification based more on what type of image is depicted.

Interestingly, while the relation between classification and liking scores cannot definitively tell us about causality, the lower correlation between score found among participants who did use a categorical basis, and higher scores among those using hedonic factors, might tend to suggest a key difference between these groups. It may be that those who do resort to hedonic assessments may be more likely to punish art that is not liked as itself not an artwork, suggesting a rating > classification direction. Whereas those who used categorical approaches may show a classification > rating order. This of course is a question for future research.

In turn, regarding attitudes and personality, our findings paint a picture of a participant, likely to agree that objects are art, who shows classic indicators of art interest. This includes believing art is important, being interested in and comfortable with art. Equally, such individuals also showed high openness to experience, low need for cognitive closure, and expectations that one should have a deep connection with artworks. It was also this group who showed a significant correlation with an experience-based motive for classification assessments. Thus, we would suggest that research consider these aspects when preparing studies and assessing participants. Note also that these individuals, while feeling themselves knowledgeable about art, did not necessarily show aspects of training or expertise. This of course may derive from our sample of lay students. However, such populations are commonly used in laboratory study. Interestingly, it also appeared that specific personalities or levels of art interest did not correlate with the propensity to use a hedonic or categorical classification basis, suggesting the complexity of this topic when attempting to further explain why individuals did not always classify images as art.

Finally, the significant connections between strategies and personality which were discovered suggest one last basis which may also be important for understanding classification. Positive predictors for classification, in addition to the categorical aspect above, involved the work's impact or the viewer's processing experience. Individuals felt that objects which evoked thoughts or emotions, or challenged them, were more likely to be art. This “processing experience”-based aspect was further supported in the Principle Component Analysis, as a collection of classification strategies which significantly associated with positive art classification. Interestingly, this basis has been previously identified as a means of determining art. For example, it aligns with arguments by Dewey (1980), that an artwork is determined by its resulting experience. Wartenberg (2006), in a philosophical review of different perspectives on defining art, also notes the “communication of feeling” as a strategy supported by for example Tolstoy (also Langer, 1953; Crittenden, 1968). In the same vein, Eaton (2000, p. 153) argued for art as primarily “interpretation delivery devices.” The previous art classification study by Hagtvedt and Patrick (2008), which again is the only other known empirical assessment of this question, also found that respondents, in free reports, noted art images as being particularly expressive or emotion-inducing.

It is again not clear whether this focus on experience spoke to more of a classificatory or hedonic motive: viewers may have believed that these factors were the main categorical determinants of a work of art (i.e., “art *is* that which provokes thoughts”), a belief which would of course be most salient in Post-Modern, Readymade or non-mimetic cases. Alternatively, viewers may have felt that art that did impact them in some way was more pleasing or good, and thus deserved to be called an artwork. This also tends to match current sociological or culturally-emergent paradigms for determining art as fulfilling certain human-centered aims. For example, as noted by Margolis (1974, p. 192) “there is nothing to be identified as a work of art [referring to the physical appearance] except relative to a cultural concern with certain patterns of purposiveness.” Thus, it may be this attitude that is required for study on Conceptual, Readymade, Postmodern, or, to a lesser extent, Abstract pieces. Certainly, this is one more target for future systematic study.

This study also comes with caveats. Although, we employed a range of art, there are still many other varieties that we did not asses and which are commonly used in present empirical research (e.g., impressionism, sculpture, etc.). It would be interesting for future studies to employ an even more assessment of multiple art styles, assessing whether the same classification patterns are discovered. Based on the present paper's argument and findings, this itself would be an important step for any lab studies before using a particular art variety. Equally, it would be interesting to consider if art experts show the same propensity for case-by-case arthood determinations (Kirk et al., 2009a; Leder et al., 2014) or respond with hedonic, categorical, or experience-based motives. Similarly, studies might consider application in different countries/cultures. Due to our art selection choices and our decision to combine results into a general art classification score for the PCAs, there may have also been some bias toward certain personality types that might have favored our artwork selection and which might show different patterns if using different mixes of art. This too could be further assessed in future research. The study, due to its exploratory character, also included a number of parallel assessments without correction. We suggest that specific relationships of interest be considered in further, controlled follow-up assessments with larger samples. Finally, because liking and classification questions were asked in tandem we cannot make direct claims about causality. This too should be further tested.

In conclusion, it does appear that typical art study viewers do not automatically assign art classification to viewed objects—even if chosen from respected art canon. This shares a strong correlation to their preference ratings. And, to return to the quote that began this paper, this result should be heeded by researchers who wish to assess the effects of “Art”: It does not matter whether the researcher, or the art community, deems a stimulus to be an artwork. Here, the participant is the master, with classifications that may underlie some or many current findings, and should be explicitly considered in psychological and empirical research.

## Author contributions

The study design, execution, data analysis and writing were done by MP and YC. PM contributed to the design, execution, and writing. GG contributed to data analysis and writing. HL assisted with design and paper writing.

### Conflict of interest statement

The authors declare that the research was conducted in the absence of any commercial or financial relationships that could be construed as a potential conflict of interest.

## Footnotes

^1^To take two examples: Modernism and the idea that art should be mimetic was challenged, among others, by Picasso and the cubists—e.g., especially see the debate surrounding the painting *Les Demoiselles d'Avignon* (Richardson, 1991). Matisse, who's own Fauvism style was threatened by the new Modernist approach, famously claimed that the Picasso painting was “hideous” (Richardson, 1991). The *Brillo Box* by Andy Warhol, questioned whether art could even be detected by the eye, or whether it had to be assumed by context (Danto, 1974). Danto's (1992, p. 37) recollection of his colleagues who “hated” Warhol's new art, and importantly, “were not ready for” art which revoked previous art-related aspects.

## References

[B1] Altman D. G. Bland J. M. (1995). Statistics notes: the normal distribution. BMJ 310:298. 10.1136/bmj.310.6975.298 7866172 PMC2548695

[B2] Arai S. Kawabata H. (2016). Appreciation contexts modulate aesthetic evaluation and perceived duration of pictures. Art Percept. 4, 225–239. 10.1163/22134913-00002052

[B3] Bailey G. W. S. (2000). Art? life after death, in Theories of Art Today, ed Carroll N. (Madison, WI: The University of Wisconsin Press), 160–174.

[B4] Becker H. S. (1982). Art Worlds. Berkeley, CA: University of California Press.

[B5] Boas G. (1943). Art and reality. Coll. Art J. 2, 115–117. 10.2307/773341

[B6] Bourdieu P. Darbel A. Schnapper D. (1997). The Love of Art: European Art Museums and their Public. Cambridge: Polity Press.

[B7] Brieber D. Nadal M. Leder H. (2015). In the white cube: museum context enhances the valuation and memory of art. Acta Psychol. 154, 36–42. 10.1016/j.actpsy.2014.11.004 25481660

[B8] Brieber D. Nadal M. Leder H. Rosenberg R. (2014). Art in time and space: context modulates the relation between art experience and viewing time. PLoS ONE 9:e99019. 10.1371/journal.pone.0099019 24892829 PMC4043844

[B9] Cela-Conde C. J. García-Prieto J. Ramasco J. J. Mirasso C. R. Bajo R. Munar E.. (2013). Dynamics of brain networks in the aesthetic appreciation. Proc. Natl. Acad. Sci. U.S.A. 110, 10454–10461. 10.1073/pnas.1302855110 23754437 PMC3690613

[B10] Chamorro-Premuzic T. Furnham A. (2004). Art judgement: a measure related to both personality and intelligence? Imagin. Cogn. Pers. 24, 3–24. 10.2190/U4LW-TH9X-80M3-NJ54

[B11] Chatterjee A. Widick P. Sternschein R. Smith W. B. Bromberger B. (2010). The assessment of art attributes. Emp. Stud. Arts 28, 207–222. 10.2190/EM.28.2.f

[B12] Chokron S. De Agostini M. (2000). Reading habits influence aesthetic preference. Cogn. Brain Res. 10, 45–49. 10.1016/S0926-6410(00)00021-5 10978691

[B13] Crittenden B. S. (1968). From description to evaluation in aesthetic judgment. J. Aesthetic Educ. 2, 37–58. 10.2307/3331644

[B14] Cupchik G. C. Vartanian O. Crawley A. Mikulis D. J. (2009). Viewing artworks: contributions of cognitive control and perceptual facilitation to aesthetic experience. Brain Cogn. 70, 84–91. 10.1016/j.bandc.2009.01.003 19223099

[B15] Currie G. (1985). The authentic and the aesthetic. Am. Philos. Q. 22, 153–160.

[B16] Danto A. C. (1974). The transfiguration of the commonplace. J. Aesthet. Art Crit. 33, 139–172. 10.2307/429082

[B17] Danto A. C. (1992). Beyond the Brillo Box; the Visual Arts in Post-Historical Perspective. New York, NY: Farrar, Straus, Giroux.

[B18] Danto A. C. (2000). Art and meaning, in Theories of Art Today, ed Carroll N. (Madison, WI: The University of Wisconsin Press), 130–140.

[B19] Dewey J. (1980). Art as Experience. New York, NY: Perigee.

[B20] Dezeuze A. (2005). Transfiguration of the commonplace. Variant 22, 17–19. Available online at: http://www.variant.org.uk/22texts/Dezeuze.html

[B21] Di Dio C. Macaluso E. Rizzolatti G. (2007). The golden beauty: brain response to classical and renaissance sculptures. PLoS ONE 2:e1201. 10.1371/journal.pone.0001201 18030335 PMC2065898

[B22] Dutton D. (2006). A naturalist definition of art. J. Aesthet. Art Crit. 64, 367–377. 10.1111/j.1540-594X.2006.00217.x

[B23] Eaton M. M. (2000). A sustainable definition of “Art”, in Theories of Art Today, ed Carroll N. (Madison, WI: The University of Wisconsin Press), 141–159.

[B24] Fayn K. MacCann C. Tiliopoulos N. Silvia P. J. (2015). Aesthetic emotions and aesthetic people: openness predicts sensitivity to novelty in the experiences of interest and pleasure. Front. Psychol. 6:1877. 10.3389/fpsyg.2015.01877 26696940 PMC4673303

[B25] Field A. (2009). Discovering Statistics Using SPSS, 3 Edn. London: SAGE publications Ltd.

[B26] Furnham A. Walker J. (2001). The influence of personality traits, previous experience of art, and demographic variables on artistic preference. Pers. Individ. Dif. 31, 997–1017. 10.1016/S0191-8869(00)00202-6

[B27] Gartus A. Leder H. (2014). The white cube of the museum versus the gray cube of the street: the role of context in aesthetic evaluations. Psychol. Aesthet. Creat. Arts 8, 311–320. 10.1037/a0036847

[B28] Gerger G. Leder H. Kremer A. (2014). Context effects on emotional and aesthetic evaluations of artworks and IAPS pictures. Acta Psychol. 151, 174–183. 10.1016/j.actpsy.2014.06.008 24983515

[B29] Giannini A. M. Tizzani E. Baralla F. Gurrieri G. (2013). What I like is how i am: impact of Alexithymia on aesthetic preference. Creat. Res. J. 25, 312–316. 10.1080/10400419.2013.813796

[B30] Goldie P. Schellekens E. (2010). Who's Afraid of Conceptual Art? London: Routledge.

[B31] Graham D. Stockinger S. Leder H. (2013). An island of stability: art images and natural scenes–but not natural faces–show consistent esthetic response in Alzheimer's-related dementia. Front. Psychol. 4:107 10.3389/fpsyg.2013.00107 23471005 PMC3590566

[B32] Hagtvedt H. Patrick V. M. (2008). Art infusion: the influence of visual art on the perception and evaluation of consumer products. J. Market. Res. 45, 379–389. 10.1509/jmkr.45.3.379

[B33] Hagtvedt H. Hagtvedt R. Patrick V. M. (2008). The perception and evaluation of visual art. Emp. Stud. Arts 26, 197–218. 10.2190/EM.26.2.d

[B34] Hanich J. Wagner V. Shah M. Jacobsen T. Menninghaus W. (2014). Why we like to watch sad films. The pleasure of being moved in aesthetic experiences. Psychol. Aesthet. Creat.Arts 8, 130–143. 10.1037/a0035690

[B35] Hanquinet L. (2013). Visitors to modern and contemporary art museums: towards a new sociology of ‘cultural profiles’. Sociol. Rev. 61, 790–813. 10.1111/1467-954X.12072

[B36] Hauser A. (1999). The Social History of Art. London: Routledge.

[B37] Henson R. K. Roberts J. K. (2006). Use of exploratory factor analysis in published research: common errors and some comment on improved practice. Educ. Psychol. Meas. 66, 393–416. 10.1177/0013164405282485

[B38] Holmes T. Zanker J. M. (2012). Using an oculomotor signature as an indicator of aesthetic preference. i-Perception 3, 426–439. 10.1068/i0448aap 23145294 PMC3485839

[B39] Howell D. C. (1997). Statistical Methods for Psychology, 4th Edn. Belmont, CA: Duxbury.

[B40] Huang M. Bridge H. Kemp M. J. Parker A. J. (2011). Human cortical activity evoked by the assignment of authenticity when viewing works of art. Front. Hum. Neurosci. 5:134. 10.3389/fnhum.2011.00134 22164139 PMC3225016

[B41] Jacobsen T. Schubotz R. I. Höfel L. Cramon D. Y. V. (2006). Brain correlates of aesthetic judgment of beauty. Neuroimage 29, 276–285. 10.1016/j.neuroimage.2005.07.010 16087351

[B42] Jakesch M. Leder H. (2015). The qualitative side of complexity: testing effects of ambiguity on complexity judgments. Psychol. Aesthet. Creat. Arts 9, 200–205. 10.1037/a0039350

[B43] Kamber R. (2011). Experimental philosophy of art. J. Aesthet. Art Crit. 69, 197–208. 10.1111/j.1540-6245.2011.01461.x

[B44] Kaufman J. C. Baer J. (2004). Sure, I'm creative—but not in mathematics!: self-reported creativity in diverse domains. Emp. Stud. Arts 22, 143–155. 10.2190/26HQ-VHE8-GTLN-BJJM

[B45] Kaufman S. B. (2013). Opening up openness to experience: A four-factor model and relations to creative achievement in the arts and sciences. J. Creat. Behav. 47, 233–255. 10.1002/jocb.33

[B46] Kaufman S. B. Quilty L. C. Grazioplene R. G. Hirsh J. B. Gray J. R. Peterson J. B. (2015). Openness to experience and intellect differentially predict creative achievement in the arts and sciences. J. Pers. 82, 248–258. 10.1111/jopy.12156 PMC4459939 25487993

[B47] Kiefer A. (2005). The intentional model in interpretation. J. Aesthet. Art Crit. 63, 271–281. 10.1111/j.0021-8529.2005.00207.x

[B48] Kirk U. Skov M. Christensen M. S. Nygaard N. (2009a). Brain correlates of aesthetic expertise: a parametric fMRI study. Brain Cogn. 69, 306–315. 10.1016/j.bandc.2008.08.004 18783864

[B49] Kirk U. Skov M. Hulme O. Christensen M. S. Zeki S. (2009b). Modulation of aesthetic value by semantic context: An fMRI study. Neuroimage 44, 1125–1132. 10.1016/j.neuroimage.2008.10.009 19010423

[B50] Kühn S. Gallinat J. (2012). The neural correlates of subjective pleasantness. Neuroimage 61, 289–294. 10.1016/j.neuroimage.2012.02.065 22406357

[B51] Lacey S. Hagtvedt H. Patrick V. M. Anderson A. Stilla R. Deshpande G.. (2011). Art for reward's sake: visual art recruits the ventral striatum. Neuroimage 55, 420–433. 10.1016/j.neuroimage.2010.11.027 21111833 PMC3031763

[B52] Langer S. K. (1953). Feeling and Form. New York, NY: Scribner.

[B53] Lauring J. O. Pelowski M. Forster M. Gondan M. Ptito M. Kupers R. (2016). Well, if they like it… Effects of social groups' ratings and price information on the appreciation of art. Psychol. Aesthet. Creat. Arts 10 344–359. 10.1037/aca0000063

[B54] Leder H. Nadal M. (2014). Ten years of a model of aesthetic appreciation and aesthetic judgments: the aesthetic episode–developments and challenges in empirical aesthetics. Br. J. Psychol. 105, 443–464. 10.1111/bjop.12084 25280118

[B55] Leder H. Belke B. Oeberst A. Augustin D. (2004). A model of aesthetic appreciation and aesthetic judgments. Br. J. Psychol. 95, 489–508. 10.1348/0007126042369811 15527534

[B56] Leder H. Gerger G. Brieber D. Schwarz N. (2014). What makes an art expert? Emotion and evaluation in art appreciation. Cogn. Emot. 28, 1137–1147. 10.1080/02699931.2013.870132 24383619

[B57] Leone L. Chirumbolo A. (2008). Conservatism as motivated avoidance of affect: need for affect scales predict conservatism measures. J. Res. Pers. 42, 755–762. 10.1016/j.jrp.2007.08.001

[B58] Leyssen M. H. Linsen S. Sammartino J. Palmer S. E. (2012). Aesthetic preference for spatial composition in multiobject pictures. Iperception 3, 25–49. 10.1068/i0458aap 23145265 PMC3485813

[B59] Liu X. Hairston J. Schrier M. Fan J. (2011). Common and distinct networks underlying reward valence and processing stages: a meta-analysis of functional neuroimaging studies. Neurosci. Biobehav. Rev. 35, 1219–1236. 10.1016/j.neubiorev.2010.12.012 21185861 PMC3395003

[B60] Locher P. Dolese M. (2004). A comparison of the perceived pictorial and aesthetic qualities of original paintings and their postcard images. Emp. Stud. Arts 22, 129–142. 10.2190/EQTC-09LF-JRHA-XKJT

[B61] Locher P. Smith J. K. Smith L. F. (2001). The influence of presentation format and viewer training in the visual arts on the perception of pictorial and aesthetic qualities of paintings. Perception 30, 449–465. 10.1068/p3008 11383192

[B62] Locher P. Smith L. F. Smith J. K. (1999). Original paintings versus slide and computer reproductions: a comparison of viewer responses. Emp. Stud. Arts 17, 121–129. 10.2190/R1WN-TAF2-376D-EFUH

[B63] Margolis J. (1974). Works of art as physically embodied and culturally emergent entities. Br. J. Aesthet. 14, 187–196. 10.1093/bjaesthetics/14.3.187

[B64] Masuda T. Gonzalez R. Kwan L. Nisbett R. E. (2008). Culture and aesthetic preference: comparing the attention to context of East Asians and Americans. Person. Soc. Psychol. Bull. 34, 1260–1275. 10.1177/0146167208320555 18678860

[B65] Muth C. Carbon C. C. (2013). The Aesthetic Aha: on the pleasure of having insights into Gestalt. Acta Psychol. 144, 25–30. 10.1016/j.actpsy.2013.05.001 23743342

[B66] Myszkowski N. Storme M. Zenasni F. Lubart T. (2014). Is visual aesthetic sensitivity independent from intelligence, personality and creativity? Pers. Individ. Dif. 59, 16–20. 10.1016/j.paid.2013.10.021

[B67] Nadal M. Munar E. Capó M. À. Rosselló J. Cela-Conde C. J. (2008). Towards a framework for the study of the neural correlates of aesthetic preference. Spat. Vis. 21, 379–396. 10.1163/156856808784532653 18534110

[B68] Noguchi Y. Murota M. (2013). Temporal dynamics of neural activity in an integration of visual and contextual information in an esthetic preference task. Neuropsychologia 51, 1077–1084. 10.1016/j.neuropsychologia.2013.03.003 23499850

[B69] Parsons M. J. (1987). How We Understand Art: A Cognitive Developmental Account of Aesthetic Experience. Cambridge: Cambridge University Press.

[B70] Pelowski M. (2015). Tears and transformation: feeling Like crying as an indicator of insightful or ‘aesthetic’ experience in empirical study of art. Front. Psychol. 6. 1–23. 10.3389/fpsyg.2015.01006 26257671 PMC4511828

[B71] Pelowski M. Akiba F. (2011). A model of art perception, evaluation and emotion in transformative aesthetic experience. New Ideas Psychol. 29, 80–97. 10.1016/j.newideapsych.2010.04.001

[B72] Pelowski M. Markey P. S. Lauring J. O. Leder H. (2016). Visualizing the impact of art: an update and comparison of current psychological models of art experience. Front. Hum. Neurosci. 10:160. 10.3389/fnhum.2016.00160 27199697 PMC4844603

[B73] Pelowski M. Markey P. S. Forster M. Gerger G. Leder H. (2017). Move me, astonish me… delight my eyes and brain: the Vienna Integrated Model of top-down and bottom-up processes in Art Perception (VIMAP) and corresponding affective, evaluative, and neurophysiological correlates. Phys. Life Rev. 21, 80–125. 10.1016/j.plrev.2017.02.003 28347673

[B74] Rammstedt B. John O. P. (2007). Measuring personality in one minute or less: a 10-item short version of the Big Five Inventory in English and German. J. Res. Pers. 41, 203–212. 10.1016/j.jrp.2006.02.001

[B75] Richardson J. (1991). A Life of Picasso, The Cubist Rebel 1907–1916. New York, NY: Alfred A. Knopf.

[B76] Roets A. Van Hiel A. (2011). Item selection and validation of a brief, 15-item version of the Need for Closure Scale. Pers. Individ. Dif. 50, 90–94. 10.1016/j.paid.2010.09.004

[B77] Rollins M. (2004). What Monet meant: Intention and attention in understanding art. J. Aesthet. Art Crit. 62, 175–188. 10.1111/j.1540-594X.2004.00150.x

[B78] Rosenberg R. Klein C. (2015). The moving eye of the beholder: eye-tracking and the perception of paintings, in Art, Aesthetics and the Brain, eds Huston J. P. Nadal M. Mora F. Agnati L. Cela-Conde C. J. (London: Oxford), 79–108.

[B79] Rothman K. J. (1990). No adjustments are needed for multiple comparisons. Epidemiology 1, 43–46. 10.1097/00001648-199001000-00010 2081237

[B80] Schlink S. Walther E. (2007). Kurz und gut: eine deutsche Kurzskala zur Erfassung des Bedürfnisses nach kognitiver Geschlossenheit. Zeitschr. Sozialpsychol. 38, 153–161. 10.1024/0044-3514.38.3.153

[B81] Schloss K. B. Hawthorne-Madell D. Palmer S. E. (2015). Ecological influences on individual differences in color preference. Attent. Percept. Psychophys. 77, 2803–2816. 10.3758/s13414-015-0954-x 26272366

[B82] Shusterman R. (2000). Pragmatist Aesthetics; Living Beauty, Rethinking Art. Lanham, MD: Rowman & Littlefield.

[B83] Silvers A. (1976). The artwork discarded. J. Aesthet. Art Crit. 34, 441–454. 10.2307/430578

[B84] Silvia P. J. (2009). Looking past pleasure: anger, confusion, disgust, pride, surprise, and other unusual aesthetic emotions. Psychol. Aesthet. Creat. Arts 3, 48–51. 10.1037/a0014632

[B85] Stecker R. (2000). Is it reasonable to attempt to define art? in Theories of Art Today, ed Carroll N. (Madison, WI: The University of Wisconsin Press), 45–64.

[B86] Tröndle M. Kirchberg V. Tschacher W. (2014). Is this art? an experimental study on visitors' judgement of contemporary art. Cult. Sociol. 8, 310–332. 10.1177/1749975513507243

[B87] Tschacher W. Greenwood S. Kirchberg V. Wintzerith S. van den Berg K. Tröndle M. (2012). Physiological correlates of aesthetic perception of artworks in a museum. Psychol. Aesthet. Creat. Arts 6:96 10.1037/a0023845

[B88] Umilta M. A. Berchio C. Sestito M. Freedberg D. Gallese V. (2012). Abstract art and cortical motor activation: an EEG study. Front. Hum. Neurosci. 6:311. 10.3389/fnhum.2012.00311 23162456 PMC3499799

[B89] Van Dongen N. N. Van Strien J. W. Dijkstra K. (2016). Implicit emotion regulation in the context of viewing artworks: ERP evidence in response to pleasant and unpleasant pictures. Brain Cogn. 107, 48–54. 10.1016/j.bandc.2016.06.003 27367861

[B90] Vartanian O. Goel V. (2004). Neuroanatomical correlates of aesthetic preference for paintings. Neuroreport 15, 893–897. 10.1097/00001756-200404090-00032 15073538

[B91] Vessel E. A. Starr G. G. Rubin N. (2012). The brain on art: intense aesthetic experience activates the default mode network. Front. Hum. Neurosci. 6:66. 10.3389/fnhum.2012.00066 22529785 PMC3330757

[B92] Wartenberg T. E. (2006). The Nature of Art: An Anthology. Belmont, CA: Wadsworth.

[B93] Wolz S. H. Carbon C. C. (2014). What's wrong with an art fake? cognitive and emotional variables influenced by authenticity status of artworks. Leonardo 47, 467–473. 10.1162/LEON_a_00869

