# Supplementary material for: But Is It really Art? The Classification of Images as “Art”/“Not Art” and Correlation with Appraisal and Viewer Interpersonal Differences
Source: Front Psychol. 2017 Oct 9;8:1729. doi: 10.3389/fpsyg.2017.01729 (PMC5640778; doi:10.3389/fpsyg.2017.01729)
Supplement: Supplementary file 1 [file Table1.docx]

**Supplementary Material**

**Table S1: List of Images**

| **Image Type** |  | **Artist** | **Title** | **Year** |
| --- | --- | --- | --- | --- |
| Abstract | 1 | Cy Twombly | untitled | 1970 |
|  | 2 | Hans Hofmann | Twilight | 1957 |
|  | 3 | Sam Gilliam | Coffee thyme | 1980 |
|  | 4 | Barnett Newman | Cathedra magna | 1951 |
|  | 5 | Herman de Vries | untitled | 2015 |
|  | 6 | Ellsworth Kelly | Cowboy | 1958 |
|  | 7 | Hans Hofmann | Oracle II | 1961 |
|  | 8 | Verena Loewensberg | untitled | 1984 |
|  | 9 | Clyfford Still | untitled | 1977 |
|  | 10 | Josef Albers | Homage to the Square: Apparition | 1959 |
|  | 11 | Carl Otto Götz | Matador (part of triptych "UDZ") | 1958 |
|  | 12 | Lucio Fontana | Concetto spaziale, Attese | 1959 |
|  | 13 | Jules Olitski | Princess Yellow | 1962 |
|  | 14 | Frank Stella | Die Fahne hoch! | 1959 |
|  | 15 | Clyfford Still | PH-379 (1950-K-No. 1) | 1950 |
|  | 16 | Cy Twombly | Untitled (Bacchus) | 2005 |
|  | 17 | Willem De Kooning | Untitled XX | 1977 |
|  | 18 | Hans Hofmann | Untitled | 1943 |
|  | 19 | Barnett Newman | Onement VI | 1953 |
|  | 20 | Herman de Vries | v74-30s | 1970-75 |
|  | 21 | José de Almada- Negreiros | Quadrante I [Quadrant I] | 1957 |
|  | 22 | Helen Frankenthaler | Only Orange | 1963 |
|  | 23 | Josef Albers | Homage to the Square | 1965 |
|  | 24 | Hans Hofmann | Astral Nebula | 1961 |
|  | 25 | Blinky Palermo | 4 Prototypen | 1970 |
|  | 26 | Cy Twombly | A Tree in Naples | 1960 |
|  | 27 | Lucio Fontana | Concetto Spaziale, Attese | 1968 |
|  | 28 | Lucia Fontana | Nudo Rosa | 1967 |
|  | 29 | Frank Stella | The Marriage of Reason and Squalor, II | 1959 |
|  | 30 | Fanz Kline | Mahoning | 1956 |
|  |  |  |  |  |
| Readymade | 31 | Angela de la Cruz | Flat | 2009 |
|  | 32 | Sara Goldschmied & Eleonora Chiari | Where Shall We Go Dancing Tonight? | 2015 |
|  | 33 | Oto Gillen | untitled (Vanitas) | 2007 |
|  | 34 | Jeff Koons | Vacuum Cleaner | 1981 |
|  | 35 | Hans-Peter Feldmann | Bread | 2008 |
|  | 36 | Anonymous | "Shoefiti" Shoes on telephone pole | unknown |
|  | 37 | Dan Flavin | Pink out of a Corner (to Jasper Johns) | 1963 |
|  | 38 | Robert Ryman | Pace | 1984 |
|  | 39 | Luis Camnitzer | Living room | 1937 |
|  | 40 | Hans Hacke | Collateral | 1991 |
|  | 41 | Tracey Emin | My Bed | 1998 |
|  | 42 | Herman de Vries | Random Object (V 75- 62) | 1975 |
|  | 43 | Baldessari | I will not make any more boring art | 1971 |
|  | 44 | Alighiero Boetti | Dama | 1968 |
|  | 45 | Cedrik Alby | L'alternative au Hérisson | 2012 |
|  | 46 | Adolf Krischanitz | Barhocker | 1986 |
|  | 47 | André Pierre Arnal | Opéra | 1975 |
|  | 48 | Daniel Spoerri | Tableau piège- Restaurant de la City Galerie, Zurich | 1965 |
|  | 49 | Marcel Duchamp | In Advance of the Broken Arm | 1915 |
|  | 50 | Joseph Beuys | Capri Batterie | 1985 |
|  | 51 | John Baldessari | Throwing Three Balls In The Air To Get A Straight Line | 1973 |
|  | 52 | Dan Flavin | The Nominal Three | 1963 |
|  | 53 | Paul Nugent | Séance | 2011 |
|  | 54 | George Maciunas | Fluxkit | 1964-65 |
|  | 55 | Bill Bollinger | Untitled | 1966 |
|  | 56 | Marcel Duchamp | Fresh Window | 1920 |
|  | 57 | Giovanni Anselmo | Direzione | 1978 |
|  | 58 | La Monte Young | Composition #7 | 1960 |
|  | 59 | Carl Andre | Equivalent V | 1966-69 |
|  | 60 | Marcel Duchamp | Porte- bouteilles (Bottle Rack) | 1914 |
|  |  |  |  |  |
| Hyperrealistic | 61 | Roberto Bernardi | Candy Machine | 2009 |
|  | 62 | Andrew Grassie | Private | unknown |
|  | 63 | Mike Bayne | Mac's | 2010 |
|  | 64 | Jeffrey T. Larson | Jars of Pasta | 2008 |
|  | 65 | Robert Bechtle | Berkeley Pinto | 1969-70 |
|  | 66 | Tom Martin | Complementary Flavours | 2010 |
|  | 67 | Jason de Graaf | A perfect day in which nothing really happened | unknown |
|  | 68 | Tom Martin | Summer Sensations | 2010 |
|  | 69 | Nathan Walsh | New York Reflections | 2009 |
|  | 70 | Mark Goings | Two-tone Terry | 2009 |
|  | 71 | Steve Mills | The tip | 2001 |
|  | 72 | Jeffrey T. Larson | Open Box | 2007 |
|  | 73 | Tom Martin | It's all good | 2009 |
|  | 74 | Jason de Graaf | Bedlam | unknown |
|  | 75 | Gregory Thielker | Coming to a complete stop | 2008 |
|  | 76 | Tom Martin | My First Time In New York | 2010 |
|  | 77 | Andrew Grassie | Private: Reception | 2006 |
|  | 78 | Mike Bayne | Oil Paintings By Robert Ayre | 2010 |
|  | 79 | Jeffrey T. Larson | Cargill Past. Nourishing People | 2007 |
|  | 80 | Malcolm Morley | Portrait Of Esses In Central Park | 1969-70 |
|  | 81 | Tom Martin | Made With Tomato | 2011 |
|  | 82 | Pedro Campos | Corn flakes | unknown |
|  | 83 | Jason de Graaf | Strawberries on foil | unknown |
|  | 84 | Nathan Walsh | Brooklyn Bridge | 2008 |
|  | 85 | Mike Bayne | Untitled (Downtown Owl) | 2010 |
|  | 86 | Jason de Graaf | Aether | 2013 |
|  | 87 | Nathan Walsh | Little Russia | 2009 |
|  | 88 | Tom Martin | Perfect Porridge | 2009 |
|  | 89 | Pedro Campos | Pencils and Freud | unknown |
|  | 90 | Gregory Thielker | unknown (Under an Unminding Sky series) | 2006-2010 |
|  |  |  |  |  |
| Bad/Kitsch | 91 | Fritz von Uhde | Die Kinderstube | 1889 |
|  | 92 | Edward Killingsworth Johnson | A new friend | 1873 |
|  | 93 | Louis Gabriel Eugene Isabey | Drei Frauen in einem Park | 1852 |
|  | 94 | Wilhelm Schwar | Der Grosse Freunde | 1904 |
|  | 95 | Thomas Kinkade | Everett's Cottage | 1997 |
|  | 96 | Léon Charles Huber | Kittens at Play | 1905 |
|  | 97 | Charles James Lewis | Reading by the Window, Hastings | 1880 |
|  | 98 | Edward Killingsworth Johnson | The New Friend | 1880 |
|  | 99 | Nicolas Lanret | A Lady and Gentleman Taking Coffee with Children in a Garden | 1742 |
|  | 100 | Wilhelm Schwar | Best of friends | 1906 |
|  | 101 | Julius Adam | Die Einquartierung | 1890 |
|  | 102 | Thomas Kinkade | The Night Before Christmas | 2004 |
|  | 103 | Ben Butcher | Mark of the Millenium | 2011 |
|  | 104 | "Richie" | untitled (woman and horse) | unknown |
|  | 105 | Anonymous | untitled (woman in fire with wings) | unknown |
|  | 106 | Anonymous | untitled (dreaming man) | |
|  | 107 | "Florence" | untitled (cat) | unknown |
|  | 108 | Anonymous | On the Shore at Sunset | 2007 |
|  | 109 | Ben Butcher | Triceratops of Justice | 2011 |
|  | 110 | Anonymous | untitled (two dogs) | unknown |
|  | 111 | Anonymous | untitled (two people) | unknown |
|  | 112 | Anonymous | untitled ("Mona Lisa") | unknown |
|  | 113 | Anonymous | childs drawing of person | unknown |
|  | 114 | Anonymous | child's drawing | unknown |
|  | 115 | Anonymous | child's drawing "Batman" | unknown |
|  | 116 | Anonymous | childs drawing of person | unknown |
|  | 117 | "Phalgun N." | child's drawing "Dog Carrying Box" | unknown |
|  | 118 | Anonymous | child's drawing of people | unknown |
|  | 119 | "Mrs. Jackson" | Peter The Kitty | unknown |
|  | 120 | Anonymous | untitled (person holding dog) | unknown |
|  |  |  |  |  |
| "Art Control" Baroque/Rennaissance | 121 | Michelangelo Merisi da Caravaggio | Vocazione de san Matteo | 1599/1600 |
|  | 122 | Johannes Vermeer | Young Woman with a Water Pitcher | 1662 |
|  | 123 | Rembrandt H. van Rijn | The Anatomy Lesson of Dr. Nicolaes Tulp | 1932 |
|  | 124 | Peter Paul Rubens | The Fall of Phaeton | 1604/ 1605 |
|  | 125 | Giovanni Bellini | Holy Allegory | 1490- 1500 |
|  | 126 | Rembrandt van Rjin | The Parable of the Rich Fool | 1627 |
|  | 127 | Casper David Friedrich | Woman at a Window | 1822 |
|  | 128 | Michelangelo Merisi da Caravaggio | The Incredulity of Saint Thomas | 1601 |
|  | 129 | Paolo de Matteis | The triumph of the Immaculate | 1710-15 |
|  | 130 | Jan van Eyck | The Madonna of Chancellor Rolin | 1435 |
|  |  |  |  |  |
| "Not Art Control" Photos of everyday objects | 131 | ---- | photo of supermarket entrance | ---- |
|  | 132 | ---- | photo of stepladder | ---- |
|  | 133 | ---- | photo of lawn mower in yard | ---- |
|  | 134 | ---- | photo of inflatable ball in pool | ---- |
|  | 135 | ---- | photo of juicer in kitchen | ---- |
|  | 136 | ---- | photo of two cars parked on street | ---- |
|  | 137 | ---- | photo of stepladder | ---- |
|  | 138 | ---- | photo of watering can on sidewalk | ---- |
|  | 139 | ---- | photo of life preserver in pool | ---- |
|  | 140 | ---- | photo of electric mixer in kitchen | ---- |

| **Table S2** |  |  |  |  |  |  |  |  |  |  |
| --- | --- | --- | --- | --- | --- | --- | --- | --- | --- | --- |
| **Factors used in making art/not art decisions: Correlation between 1-7 answer to “how important was the following factor in making your art/not art decisions” with Percentage of objects classified as art. Pearson product-moment.** | | | | | | | | |  |  |
|  |  |  |  | Correlations | | | | | | |
|  | M | SD |  | All (non Controls) | Abstract paintings | Readymade sculpture | Hyperreal paintings | Kitsch/  Bad paintings | Renaissance/ Baroque Painting Control | Control everyday objects |
| beauty | 4.11 | 2.01 |  | **-.215^*^** | **-.260^**^** | -.182 | -.0260 | **-.235^*^** | .085 | -.107 |
| technical quality | 4.69 | 1.71 |  | **-.196^*^** | **-.218^*^** | **-.199^*^** | -.047 | -.170 | .036 | -.146 |
| evidence of making | 4.77 | 1.94 |  | -.115 | -.163 | -.122 | -.149 | .109 | .178 | -.031 |
| content | 4.60 | 1.86 |  | -.100 | -.127 | -.044 | -.022 | -.148 | .134 | .019 |
| artwork style | 5.53 | 1.50 |  | **-.196^*^** | -.110 | **-.207^*^** | **-.196^*^** | -.106 | **.211*** | **-.221*** |
| composition | 5.15 | 1.60 |  | .027 | -.061 | .075 | .133 | -.093 | **.253**** | -.005 |
| form | 3.61 | 1.71 |  | .095 | .090 | .135 | .056 | .010 | .124 | .050 |
| colors or contrast | 4.01 | 1.71 |  | .121 | .084 | .142 | .120 | .028 | .161 | .080 |
| materials | 3.69 | 1.72 |  | -.011 | -.049 | .032 | -.003 | -.023 | **.197*** | .095 |
| expensive looking | 2.38 | 1.62 |  | -.132 | -.130 | -.087 | -.091 | -.129 | .078 | -.049 |
|  |  |  |  |  |  |  |  |  |  |  |
| evokes nostalgia | 3.23 | 1.77 |  | .045 | .061 | .052 | -.011 | .045 | .127 | .019 |
| challenges me | 3.94 | 1.93 |  | .136 | .181 | .160 | .036 | .052 | .006 | .083 |
| makes me uncomfortable | 2.41 | 1.63 |  | **.207^*^** | **.219^*^** | **.241^*^** | .123 | .062 | .047 | .151 |
| makes me safe, comfortable | 3.16 | 1.85 |  | .058 | -.009 | .047 | .064 | .096 | .016 | .122 |
| novelty | 3.94 | 1.88 |  | .095 | .137 | .116 | .044 | -.008 | -.043 | .046 |
| aligns with beliefs, values | 2.78 | 1.99 |  | .022 | .068 | .016 | -.031 | .021 | -.078 | .088 |
| emotionally evocative | 4.95 | 1.85 |  | .134 | .135 | .129 | .114 | .041 | .014 | .010 |
| thought-provoking | 5.06 | 1.85 |  | **.204^*^** | **.265^**^** | **.223^*^** | .16 | -.027 | .139 | .032 |
| felt a deeper meaning | 4.59 | 1.84 |  | .045 | .031 | .093 | .017 | -.011 | .157 | -.019 |
| had no meaning, purpose | 2.16 | 1.70 |  | .124 | .109 | .175 | -.025 | .156 | .135 | .040 |
|  |  |  |  |  |  |  |  |  |  |  |
| I thought of experts' opinion | 2.63 | 1.79 |  | -.179 | -.100 | -.108 | **-.223^*^** | -.151 | .105 | **-.191*** |
| made me see the world through artist's eye | 3.73 | 1.88 |  | .158 | .130 | **.216*** | .125 | .006 | .032 | .081 |
|  |  |  |  |  |  |  |  |  |  |  |
| Every painting is automatically art | 2.66 | 1.82 |  | **.395**** | **.270**** | **.331**** | **.286**** | .**420**** | .107 | **.322**** |

*Note.* Results based on N = 114 (80 female, M age = 23.2, native German speaking) psychology students from the University of Vienna. Correlations are the result of individual Pearson Product Moment analyses (two tailed). Note also that reported correlations are uncorrected for multiple comparisons.

| **Table S3** |  |  |  |  |  |  |  |  |  |
| --- | --- | --- | --- | --- | --- | --- | --- | --- | --- |
| **Correlation with classifying objects as art: Art training, education and involvement. Pearson product-moment.** | | | | | | | |  |  |
|  | All (non control) (% art) | Abstract | Ready-made | Hyper-real | Kitsch/Bad painting | Control: Renaissance/ Baroque Painting | Control: everyday objects |  | Liking (all art) |
| **Art training and education (Chatterjee et al., 2010)** | | | |  |  |  |  |  |  |
| number of studio art classes (H.S.+) | .171 | **.233^*^** | .160 | .142 | -.016 | .013 | -.056 |  | .107 |
| number of art history classes (H.S.+) | .178 | **.200^*^** | .176 | .118 | .062 | -.014 | .043 |  | .069 |
| number of art theory or aesthetics classes (H.S.+) | .141 | **.191^*^** | .161 | .090 | -.014 | -.023 | .024 |  | -.078 |
| hours spent making visual art | .126 | .020 | .069 | .186 | .140 | -.004 | **.207^*^** |  | -.055 |
|  |  |  |  |  |  |  |  |  |  |
| **Objective art involvement (Leder et al. 2014)** | | | |  |  |  |  |  |  |
| how often visit art museums? | **.306^**^** | .**312^**^** | **.313^**^** | **.216^*^** | .121 | .037 | .167 |  | .020 |
| how often read art books? | **.309^**^** | **.336^**^** | **.298^**^** | **.188^*^** | .155 | -.070 | .136 |  | -.005 |
| how often look at pictures of art? | .129 | .168 | .140 | .088 | -.003 | .066 | .017 |  | -.041 |
| how often visit art events (lectures, etc.) | **.292^**^** | **.281^**^** | **.283^**^** | **.214^*^** | .144 | -.055 | **.273^**^** |  | .034 |
|  | | | | | | | |  |  |
| **Art knowledge and comfort (Pelowski, 2015)** | | |  |  |  |  |  |  |  |
| I am comfortable looking at and discussing art. | **.365^**^** | **.456^**^** | **.356^**^** | **.202^*^** | .133 | .136 | .073 |  | .041 |
| I am knowledgeable about art | **.379^**^** | **.421^**^** | **.352^**^** | **.247^**^** | .181 | .019 | .081 |  | .056 |
| art is important | **.417^**^** | **.442^**^** | **.386^**^** | **.288^**^** | **.207^*^** | .106 | .090 |  | .103 |
| I enjoy being challenged by art | **.440^**^** | **.498^**^** | **.432^**^** | **.285^**^** | .169 | .094 | .146 |  | .111 |
| I am interested in art | **.455^**^** | **.551^**^** | **.409^**^** | **.275^**^** | **.203^*^** | .149 | .098 |  | .072 |

*Note.* Results based on N = 114 (80 female, M age = 23.2, native German speaking) psychology students from the University of Vienna. Correlations are the result of individual Pearson Product Moment analyses (two tailed). Correlations are uncorrected for multiple comparisons.

| **Table S4** |  |  |  |  |  |  |  |  |  |
| --- | --- | --- | --- | --- | --- | --- | --- | --- | --- |
| **Correlation with classifying objects as art: General Artwork beliefs and preferences. Pearson product-moment.** | | | | | | | |  |  |
|  | All (non control) (% art) | Abstract | Ready- made | Hyper-real | Kitsch/Bad painting | Control: Renaissance/ Baroque Painting | Control: everyday objects |  | Liking (all art) |
| The best art is difficult or challenging | .032 | -.070 | .027 | .133 | .003 | .038 | **.242^**^** |  | .031 |
| The best art makes you feel | .095 | .019 | .078 | .062 | .168 | .137 | .034 |  | **.214*** |
| The best art makes you think | .072 | .006 | .093 | .129 | -.016 | .124 | .088 |  | .063 |
| The best art primarily is pleasurable | **-.265^**^** | **-.371^**^** | **-.226^*^** | -.114 | -.138 | -.060 | -.083 |  | -.027 |
| The best art should make you feel tranquil or harmony | **-.265^**^** | **-.359^**^** | **-.232^*^** | -.137 | -.105 | -.052 | -.092 |  | -.034 |
| The best art should make you feel insight | -.148 | -.136 | -.059 | -.141 | -.156 | .120 | -.126 |  | -.096 |
| The best art should make you feel Catharsis or relief | -.049 | -.104 | .018 | -.036 | -.043 | -.018 | -.061 |  | .046 |
| The best art should make you feel transformation or personal change | .105 | .080 | .176 | .158 | .054 | .086 | .086 |  | .107 |
| The best art should make you feel disrupted or uncomfortable | **.187^*^** | .158 | **.237^*^** | .149 | .029 | .028 | .118 |  | **.193*** |
| The best art should make you feel surprise | **.202^*^** | .129 | **.250^**^** | .164 | .089 | .038 | .073 |  | **.189*** |
| The best art should make you feel curiosity | .169 | .132 | **.190^*^** | **.209^*^** | -.025 | .157 | .050 |  | .143 |
| The best art should make you feel a sense of novelty | .067 | .069 | .120 | .054 | -.055 | .084 | .055 |  | .125 |
|  |  |  |  |  |  |  |  |  |  |
| the more realistic the painting, the better the artist | **-.365^**^** | **-.491^**^** | **-.351^**^** | -.098 | **-.234^*^** | .013 | -.061 |  | -.175 |
| Anybody could produce abstract art | -.106 | **-.259^**^** | -.134 | -.01 | .101 | .010 | .074 |  | -.003 |
| Everyone who can draw something realistically is a good artist | -.004 | -.140 | -.017 | .057 | .111 | **.192^*^** | .048 |  | .082 |

*Note.* Results based on N = 114 (80 female, M age = 23.2, native German speaking) psychology students from the University of Vienna. Correlations are the result of individual Pearson Product Moment analyses (two tailed). Note also that reported correltions are uncorrected for multiple comparisons.

| **Table S5** |  |  |  |  |  |  |  |  |  |
| --- | --- | --- | --- | --- | --- | --- | --- | --- | --- |
| **Correlation with classifying objects as art: Art-type preference** | | | | | | | | | |
|  | All (non control) (% art) | Abstract | Ready- made | Hyper- real | Kitsch/Bad painting/ drawing | Control: Renaissance/ Baroque Painting | Control: everyday objects |  | Liking (all art) |
| Abstract | **.329^**^** | **.421^**^** | **.301^**^** | **.214^*^** | .089 | .068 | .088 |  | .029 |
| Readymade | **.289^**^** | **.366^**^** | **.332^**^** | .101 | .113 | .124 | .030 |  | **.219*** |
| Classic | -.095 | -.149 | -.092 | -.059 | .008 | .171 | -.091 |  | -.134 |
| Kitsch | .033 | .090 | -.036 | -.083 | .177 | .050 | .031 |  | .106 |
| Avant Garde | **.499^**^** | **.544^**^** | **.494^**^** | **.377^**^** | .148 | .090 | .189 |  | **.228**** |
| Representational | .182 | .174 | .131 | .141 | .150 | .035 | -.041 |  | .054 |
| Fantasy | .053 | -.004 | -.012 | .028 | **.200^*^** | .020 | -.071 |  | .144 |
| Graffiti | **.252^**^** | **.236^*^** | .167 | .166 | **.270^**^** | -.008 | .074 |  | **.259**** |
| Digital art | **.233^*^** | .170 | .159 | **.251^**^** | .176 | .012 | .084 |  | **.275**** |
| Impressionism | .068 | .063 | .016 | .077 | .071 | .104 | .044 |  | -.029 |
| Cubism | **.201^*^** | **.226^*^** | .130 | .160 | .137 | **.208*** | .019 |  | -.078 |
| Surrealism | **.323^**^** | **.361^**^** | **.305^**^** | .184 | .178 | .185 | .008 |  | .175 |
| Pop art | **.264^**^** | **.375^**^** | **.222^*^** | .132 | .108 | -.048 | .023 |  | **.253**** |
| Conceptual art | **.234^*^** | **.304^**^** | **.280^**^** | .071 | .082 | .083 | -.005 |  | .018 |

*Note.* Results based on N = 114 (80 female, M age = 23.2, native German speaking) psychology students from the University of Vienna. Correlations are the result of individual Pearson Product Moment analyses (two tailed). Correlations are uncorrected for multiple comparisons.

| **Table S6** |  |  |  |  |  |  |  |  |  |
| --- | --- | --- | --- | --- | --- | --- | --- | --- | --- |
| **Correlation with classifying art: Expectations for visiting art/museum (from Tröndle, Kirchberg & Tschacher, 2014). Pearson product-moment.** | | | | | | | | | |
|  | All (non control) (% art) | Abstract | Ready- made | Hyper- real | Kitsch/ Bad | Control: Renaissance/ Baroque Painting | Control: everyday objects |  | Liking (all art) |
| have my thoughts provoked | .141 | .146 | .179 | .128 | -.034 | .170 | .096 |  | .183 |
| art design to be convincing | .025 | .048 | -.003 | .077 | -.059 | -.021 | .017 |  | .083 |
| enjoy silence of museum space | .044 | .092 | .053 | .008 | -.022 | .141 | .008 |  | .078 |
| Improve understanding of arts | .146 | **.204^*^** | .154 | .051 | .049 | **.190^*^** | .068 |  | .026 |
| have a nice time with family/friends | -.121 | -.115 | -.107 | -.061 | -.111 | .097 | -.026 |  | -.028 |
| be part of the art exhibitions with all my senses | .111 | .162 | .123 | .079 | -.036 | .080 | .009 |  | .081 |
| Experience deep connection to art | **.229^*^** | **.269^**^** | **.224^*^** | .124 | .110 | .067 | .105 |  | .113 |
| see something familiar which I already know | .123 | **.204^*^** | .100 | .026 | .062 | .103 | .030 |  | .002 |
| experience the beauty of artworks | -.020 | -.057 | -.028 | .021 | .003 | .052 | .017 |  | -.047 |
| be entertained | -.025 | -.031 | -.030 | -.015 | .000 | .081 | -.016 |  | -.139 |
| be surprised | **.193^*^** | **.245^**^** | **.229^*^** | .117 | -.003 | .139 | .027 |  | .019 |
| see famous artworks | **-.202^*^** | **-.238^*^** | -.144 | -.127 | -.147 | **.272^**^** | .043 |  | -.258* |

*Note.* Results based on N = 114 (80 female, M age = 23.2, native German speaking) psychology students from the University of Vienna. Correlations are the result of individual Pearson Product Moment analyses (two tailed). Correlations are uncorrected for multiple comparisons.

| **Table S7** |  |  |  |  |  |  |  |  |  |
| --- | --- | --- | --- | --- | --- | --- | --- | --- | --- |
| **Correlation with classifying objects as art: personality measures. Pearson product-moment.** | | | | | | | | | |
|  | All (non control) (% art) | Abstract | Ready- made | Hyper- real | Kitsch/ Bad | Control: Renaissance/ Baroque Painting | Control: everyday objects |  | Liking (all art) |
| BFI Extraversion | -.180 | -.089 | **-.197^*^** | -.154 | -.139 | -.104 | **-.188^*^** |  | -.161 |
| BFI Agreeableness | -.025 | -.038 | -.064 | .015 | .019 | -.027 | -.068 |  | .041 |
| BFI Conscientiousness | -.057 | -.124 | -.079 | .031 | -.006 | **.251^**^** | .040 |  | .102 |
| BFI Neuroticism | .013 | -.048 | .035 | -.001 | .066 | .114 | .005 |  | .072 |
| BFI Openness | **.380^**^** | **.403^**^** | **.351^**^** | **.256^**^** | **.196^*^** | .068 | .046 |  | .002 |
|  |  |  |  |  |  |  |  |  |  |
| Need for Cog. Closure | **-.191^*^** | **-.203^*^** | **-.200^*^** | **-.193^*^** | .025 | .035 | -.086 |  | .190 |
| Creative personality | .123 | .050 | .096 | .192 | .049 | .075 | .141 |  | -.076 |

*Note.* Results based on N = 114 (80 female, M age = 23.2, native German speaking) psychology students from the University of Vienna. Correlations are the result of individual Pearson Product Moment analyses (two tailed). Correlations are uncorrected for multiple comparisons.

| **Table S8 (1 of 2)** |  |  |  |  |  |  |  |  |  |
| --- | --- | --- | --- | --- | --- | --- | --- | --- | --- |
| **Correlation with classifying objects as art: Hanquinet (2014) Social profile of tastes and interests. Pearson product-moment.** | | | | | | | |  |  |
|  | All (non control) (% art) | Abstract | Ready- made | Hyper- real | Kitsch/Bad | Control: Renaissance/ Baroque Painting | Control: everyday objects |  | Liking (all art) |
| **Taste in music** |  |  |  |  |  |  |  |  |  |
| prefer opera, classical music | -.048 | -.133 | .018 | .062 | -.133 | -.106 | -.039 |  | -.069 |
| jazz | .109 | .062 | .103 | .115 | .069 | .087 | .053 |  | -.062 |
| electronic, dance | **.206^*^** | **.268^**^** | **.200^*^** | .138 | .034 | -.014 | -.019 |  | .225* |
| hard rock | .096 | .059 | .034 | .073 | .169 | .102 | .027 |  | .106 |
| pop | **-.188^*^** | -.155 | **-.252^**^** | -.13 | -.041 | .016 | -.067 |  | .049 |
| world music | .051 | .067 | .016 | .001 | .098 | .082 | .038 |  | -.013 |
| folk | .012 | .021 | .034 | -.084 | .083 | .082 | -.011 |  | .054 |
| Schlager | -.010 | -.009 | -.103 | .004 | .112 | -.054 | .048 |  | .081 |
|  |  |  |  |  |  |  |  |  |  |
| **Taste in books** |  |  |  |  |  |  |  |  |  |
| prefer reading practical books (e.g. cooking) | -.040 | -.008 | -.047 | -.009 | -.074 | -.020 | -.152 |  | -.014 |
| detective novels, comics | -.081 | -.149 | -.082 | .020 | -.052 | .042 | .018 |  | -.023 |
| classical literature | .071 | .063 | .126 | .037 | -.019 | .187^*^ | -.027 |  | -.062 |
| history books, non fiction | .098 | .084 | .049 | .084 | .112 | .021 | -.029 |  | .058 |
| art books | **.267^**^** | **.316^**^** | **.239^*^** | .127 | .177 | -.100 | .100 |  | .042 |
| essays | **.347^**^** | **.281^**^** | **.343^**^** | **.267^**^** | **.213^*^** | -.011 | .183 |  | .157 |

*Note.* Results based on N = 114 (80 female, M age = 23.2, native German speaking) psychology students from the University of Vienna. Correlations are the result of individual Pearson Product Moment analyses (two tailed). Correlations are uncorrected for multiple comparisons.

| **Table S8 (Continued, 2 of 2)** | | | | |  | |  | |  |
| --- | --- | --- | --- | --- | --- | --- | --- | --- | --- |
| **Correlation with classifying objects as art: Hanquinet (2014) Social profile of tastes and interests. Pearson product-moment.** | | | | | | | | |  |
|  | All (non control) (% art) | Abstract | Ready- made | Hyper- real | Kitsch/ Bad | Control: Renaissance/ Baroque Painting | Control: everyday objects |  | Liking (all art) |
| **Highbrow activities** |  |  |  |  |  |  |  |  |  |
| I have gone to theatre | .004 | .028 | .035 | .069 | -.161 | -.173 | .005 |  | .130 |
| concerts of classical music or jazz | .118 | .114 | .126 | .064 | .074 | -.010 | .038 |  | -.074 |
| dance performance | -.041 | -.053 | -.066 | -.017 | .015 | -.017 | .006 |  | .024 |
| opera | .118 | .081 | .140 | .096 | .052 | .046 | .106 |  | .099 |
| commercial art galleries | **.261^**^** | **.267^**^** | **.244^**^** | **.193^*^** | .123 | -.033 | .168 |  | .060 |
| contemporary art centers | **.251^**^** | **.237^*^** | **.247^**^** | **.214^*^** | .088 | -.035 | .158 |  | .070 |
| museums, art exhibitions | **.219^*^** | **.220^*^** | **.213^*^** | .167 | .088 | .017 | .164 |  | .088 |
| ballet | .000 | -.098 | -.037 | .061 | .096 | .079 | .030 |  | .123 |
|  |  |  |  |  |  |  |  |  |  |
| **Creative activities** |  |  |  |  |  |  |  |  |  |
| I have participated in dance | .153 | .161 | .076 | .086 | **.195^*^** | .075 | .060 |  | .075 |
| theatre | .069 | .040 | .034 | .049 | .118 | .063 | .075 |  | .117 |
| photography | **.385^**^** | **.237^*^** | **.319^**^** | **.360^**^** | **.340^**^** | .210^*^ | .229^*^ |  | .199* |
| painting/ drawing | **.284^**^** | .140 | **.255^**^** | **.274^**^** | **.252^**^** | -.039 | .254^**^ |  | .053 |
| playing music | .107 | -.065 | .093 | .102 | **.251^**^** | .030 | .126 |  | .113 |
| writing | .129 | .129 | .112 | .067 | .112 | .064 | .098 |  | .056 |
|  |  |  |  |  |  |  |  |  |  |
| **Leisure activities** |  |  |  |  |  |  |  |  |  |
| I have visited friends, family | .085 | .119 | .053 | .092 | -.002 | -.043 | .028 |  | .093 |
| watched TV | .075 | -.007 | .059 | .126 | .065 | -.040 | .151 |  | .143 |
| read a book | -.006 | -.030 | .033 | -.005 | -.024 | .211^*^ | -.002 |  | .002 |
| done odd jobs (e.g. gardening, fixing something in the house) | -.038 | .001 | -.063 | -.022 | -.036 | .054 | .044 |  | -.069 |
| gone out to eat (dinner) | .079 | .173 | .115 | .015 | -.080 | -.139 | .063 |  | .024 |
| played sports | -.081 | -.084 | -.066 | -.051 | -.063 | .076 | -.071 |  | -.139 |
| listened to the radio, music | -.034 | -.011 | -.007 | -.002 | -.109 | .115 | -.082 |  | .010 |
| gone to the cinema | .042 | .094 | .080 | -.043 | -.004 | -.126 | .016 |  | -.036 |
| attended a sporting event | -.016 | .011 | -.071 | -.044 | .079 | -.083 | .010 |  | .043 |
| played a board or video game | .113 | .000 | .100 | .108 | .174 | .002 | .156 |  | -.032 |
|  |  |  |  |  |  |  |  |  |  |
| **Purchase of art** |  |  |  |  |  |  |  |  |  |
| have purchased genuine art | .099 | .122 | .073 | .058 | .064 | -.006 | .011 |  | -.054 |
| purchased an art reproduction | .161 | **.214^*^** | .102 | .106 | .094 | -.085 | -.006 |  | -.044 |
| purchased an art book | **.255^**^** | **.209^*^** | **.299^**^** | **.223^*^** | .049 | -.080 | .173 |  | .076 |

*Note.* Results based on N = 114 (80 female, M age = 23.2, native German speaking) psychology students from the University of Vienna. Correlations are the result of individual Pearson Product Moment analyses (two tailed). Correlations are uncorrected for multiple comparisons.
